# Causal Effect of Plasminogen Activator Inhibitor Type 1 on Coronary Heart Disease

**DOI:** 10.1161/JAHA.116.004918

**Published:** 2017-05-26

**Authors:** Ci Song, Stephen Burgess, John D. Eicher, CHARGE Consortium Hemostatic Factor Working Group, ICBP Consortium, CHARGE Consortium Subclinical Working Group, Christopher J. O'Donnell, Andrew D. Johnson, Jie Huang, Maria Sabater‐Lleal, Folkert W. Asselbergs, David Tregouet, So‐Youn Shin, Jingzhong Ding, Jens Baumert, Tiphaine Oudot‐Mellakh, Lasse Folkersen, Nicholas L. Smith, Scott M. Williams, Mohammad A. Ikram, Marcus E. Kleber, Diane M. Becker, Vinh Truong, Josyf C. Mychaleckyj, Weihong Tang, Qiong Yang, Bengt Sennblad, Jason H. Moore, Frances M. K. Williams, Abbas Dehghan, Günther Silbernagel, Elisabeth M. C. Schrijvers, Shelly Smith, Mahir Karakas, Geoffrey H. Tofler, Angela Silveira, Gerjan J. Navis, Kurt Lohman, Ming‐Huei Chen, Annette Peters, Anuj Goel, Jemma C. Hopewell, John C. Chambers, Danish Saleheen, Per Lundmark, Bruce M. Psaty, Rona J. Strawbridge, Bernhard O. Boehm, Angela M. Carter, Christa Meisinger, John F. Peden, Joshua C. Bis, Barbara McKnight, John Öhrvik, Kent Taylor, Maria Grazia Franzosi, Udo Seedorf, Rory Collins, Anders Franco‐Cereceda, Ann‐Christine Syvänen, Alison H. Goodall, Lisa R. Yanek, Mary Cushman, Martina Müller‐Nurasyid, Aaron R. Folsom, Saonli Basu, Nena Matijevic, Wiek H. van Gilst, Jaspal S. Kooner, Albert Hofman, John Danesh, Robert Clarke, James B. Meigs, Sekar Kathiresan, Muredach P. Reilly, Norman Klopp, Tamara B. Harris, Bernhard R. Winkelmann, Peter J. Grant, Hans L. Hillege, Hugh Watkins, Timothy D. Spector, Lewis C. Becker, Russell P. Tracy, Winfried März, Andre G. Uitterlinden, Per Eriksson, Francois Cambien, Pierre‐Emmanuel Morange, Wolfgang Koenig, Nicole Soranzo, Pim van der Harst, Yongmei Liu, Anders Hamsten, Georg B. Ehret, Patricia B. Munroe, Kenneth M. Rice, Murielle Bochud, Andrew D. Johnson, Daniel I. Chasman, Albert V. Smith, Martin D. Tobin, Germaine C. Verwoert, Shih‐Jen Hwang, Vasyl Pihur, Peter Vollenweider, Paul F. O'Reilly, Najaf Amin, Jennifer L. Bragg‐Gresham, Alexander Teumer, Nicole L. Glazer, Lenore Launer, Jing Hua Zhao, Yurii Aulchenko, Simon Heath, Siim Sõber, Afshin Parsa, Jian'an Luan, Pankaj Arora, Abbas Dehghan, Feng Zhang, Gavin Lucas, Andrew A. Hicks, Anne U. Jackson, John F. Peden, Toshiko Tanaka, Sarah H. Wild, Igor Rudan, Wilmar Igl, Yuri Milaneschi, Alex N. Parker, Cristiano Fava, John C. Chambers, Ervin R. Fox, Meena Kumari, Min Jin Go, Pim van der Harst, Wen Hong Linda Kao, Marketa Sjögren, D. G. Vinay, Myriam Alexander, Yasuharu Tabara, Sue Shaw‐Hawkins, Peter H. Whincup, Yongmei Liu, Gang Shi, Johanna Kuusisto, Bamidele Tayo, Mark Seielstad, Xueling Sim, Khanh‐Dung Hoang Nguyen, Terho Lehtimäki, Giuseppe Matullo, Ying Wu, Tom R. Gaunt, N. Charlotte Onland‐Moret, Matthew N. Cooper, Carl G. P. Platou, Elin Org, Rebecca Hardy, Santosh Dahgam, Jutta Palmen, Veronique Vitart, Peter S. Braund, Tatiana Kuznetsova, Cuno S. P. M. Uiterwaal, Adebowale Adeyemo, Walter Palmas, Harry Campbell, Barbara Ludwig, Maciej Tomaszewski, Ioanna Tzoulaki, Nicholette D. Palmer, Thor Aspelund, Melissa Garcia, Yen‐Pei C. Chang, Jeffrey R. O'Connell, Nanette I. Steinle, Diederick E. Grobbee, Dan E. Arking, Sharon L. Kardia, Alanna C. Morrison, Dena Hernandez, Samer Najjar, Wendy L. McArdle, David Hadley, Morris J. Brown, John M. Connell, Aroon D. Hingorani, Ian N. M. Day, Debbie A. Lawlor, John P. Beilby, Robert W. Lawrence, Robert Clarke, Jemma C. Hopewell, Halit Ongen, Albert W. Dreisbach, Yali Li, J. Hunter Young, Joshua C. Bis, Mika Kähönen, Jorma Viikari, Linda S. Adair, Nanette R. Lee, Ming‐Huei Chen, Matthias Olden, Cristian Pattaro, Judith A. Hoffman Bolton, Anna Köttgen, Sven Bergmann, Vincent Mooser, Nish Chaturvedi, Timothy M. Frayling, Muhammad Islam, Tazeen H. Jafar, Jeanette Erdmann, Smita R. Kulkarni, Stefan R. Bornstein, Jürgen Grässler, Leif Groop, Benjamin F. Voight, Johannes Kettunen, Philip Howard, Andrew Taylor, Simonetta Guarrera, Fulvio Ricceri, Valur Emilsson, Andrew Plump, Inês Barroso, Kay‐Tee Khaw, Alan B. Weder, Steven C. Hunt, Yan V. Sun, Richard N. Bergman, Francis S. Collins, Lori L. Bonnycastle, Laura J. Scott, Heather M. Stringham, Leena Peltonen, Markus Perola, Erkki Vartiainen, Stefan‐Martin Brand, Jan A. Staessen, Thomas J. Wang, Paul R. Burton, Maria Soler Artigas, Yanbin Dong, Harold Snieder, Xiaoling Wang, Haidong Zhu, Kurt K. Lohman, Megan E. Rudock, Susan R. Heckbert, Nicholas L. Smith, Kerri L. Wiggins, Ayo Doumatey, Daniel Shriner, Gudrun Veldre, Margus Viigimaa, Sanjay Kinra, Dorairaj Prabhakaran, Vikal Tripathy, Carl D. Langefeld, Annika Rosengren, Dag S. Thelle, Anna Maria Corsi, Andrew Singleton, Terrence Forrester, Gina Hilton, Colin A. McKenzie, Tunde Salako, Naoharu Iwai, Yoshikuni Kita, Toshio Ogihara, Takayoshi Ohkubo, Tomonori Okamura, Hirotsugu Ueshima, Satoshi Umemura, Susana Eyheramendy, Thomas Meitinger, H.‐Erich Wichmann, Yoon Shin Cho, Hyung‐Lae Kim, Jong‐Young Lee, James Scott, Joban S. Sehmi, Weihua Zhang, Bo Hedblad, Peter Nilsson, George Davey Smith, Andrew Wong, Narisu Narisu, Alena Stančáková, Leslie J. Raffel, Jie Yao, Sekar Kathiresan, Stephen M. Schwartz, M. Arfan Ikram, W. T. Longstreth, Thomas H. Mosley, Sudha Seshadri, Nick R. G. Shrine, Louise V. Wain, Mario A. Morken, Amy J. Swift, Jaana Laitinen, Inga Prokopenko, Paavo Zitting, Jackie A. Cooper, Steve E. Humphries, John Danesh, Asif Rasheed, Anuj Goel, Anders Hamsten, Hugh Watkins, Stephan J. L. Bakker, Wiek H. van Gilst, Charles S. Janipalli, K. Radha Mani, Chittaranjan S. Yajnik, Albert Hofman, Francesco U. S. Mattace‐Raso, Ben A. Oostra, Ayse Demirkan, Aaron Isaacs, Fernando Rivadeneira, Edward G. Lakatta, Marco Orru, Angelo Scuteri, Mika Ala‐Korpela, Antti J. Kangas, Leo‐Pekka Lyytikäinen, Pasi Soininen, Taru Tukiainen, Peter Würtz, Rick Twee‐Hee Ong, Marcus Dörr, Heyo K. Kroemer, Uwe Völker, Henry Völzke, Pilar Galan, Serge Hercberg, Mark Lathrop, Diana Zelenika, Panos Deloukas, Massimo Mangino, Tim D. Spector, Guangju Zhai, James F. Meschia, Michael A. Nalls, Pankaj Sharma, Janos Terzic, M. V. Kranthi Kumar, Matthew Denniff, Ewa Zukowska‐Szczechowska, Lynne E. Wagenknecht, F. Gerald R. Fowkes, Fadi J. Charchar, Peter E. H. Schwarz, Caroline Hayward, Xiuqing Guo, Charles Rotimi, Michiel L. Bots, Eva Brand, Nilesh J. Samani, Ozren Polasek, Philippa J. Talmud, Fredrik Nyberg, Diana Kuh, Maris Laan, Kristian Hveem, Lyle J. Palmer, Yvonne T. van der Schouw, Juan P. Casas, Karen L. Mohlke, Paolo Vineis, Olli Raitakari, Santhi K. Ganesh, Tien Y. Wong, E. Shyong Tai, Richard S. Cooper, Markku Laakso, Dabeeru C. Rao, Tamara B. Harris, Richard W. Morris, Anna F. Dominiczak, Mika Kivimaki, Michael G. Marmot, Tetsuro Miki, Danish Saleheen, Giriraj R. Chandak, Josef Coresh, Gerjan Navis, Veikko Salomaa, Bok‐Ghee Han, Xiaofeng Zhu, Jaspal S. Kooner, Olle Melander, Paul M. Ridker, Stefania Bandinelli, Ulf B. Gyllensten, Alan F. Wright, James F. Wilson, Luigi Ferrucci, Martin Farrall, Jaakko Tuomilehto, Peter P. Pramstaller, Roberto Elosua, Nicole Soranzo, Eric J. G. Sijbrands, David Altshuler, Ruth J. F. Loos, Alan R. Shuldiner, Christian Gieger, Pierre Meneton, Andre G. Uitterlinden, Nicholas J. Wareham, Vilmundur Gudnason, Jerome I. Rotter, Rainer Rettig, Manuela Uda, David P. Strachan, Jacqueline C. M. Witteman, Anna‐Liisa Hartikainen, Jacques S. Beckmann, Eric Boerwinkle, Ramachandran S. Vasan, Michael Boehnke, Martin G. Larson, Marjo‐Riitta Järvelin, Bruce M. Psaty, Gonçalo R. Abecasis, Aravinda Chakravarti, Paul Elliott, Cornelia M. van Duijn, Christopher Newton‐Cheh, Daniel Levy, Mark J. Caulfield, Toby Johnson, Aad van der Lugt, Aaron Isaacs, Abbas Dehghan, Afshin Parsa, Alan R. Shuldiner, Albert Hofman, Albert V. Smith, Aldi T. Kraja, Andre G. Uitterlinden, Andre Uitterlinden, Andreas Ziegler, Andrew D. Johnson, Angelo Scuteri, Anne B. Newman, Arne Schillert, Benjamin F. Voight, Ben Oostra, Bolli Thorsson, Braxton D. Mitchell, Bruce M. Psaty, Caroline Hayward, Caroline S. Fox, Charles C. White, Christa Meisinger, Christie Ballantyne, Cornelia van Duijn, David Altshuler, David M. Herrington, Daniel H. O'Leary, David S. Siscovick, David J. Couper, Edward G. Lakatta, Eran Halperin, Eric Boerwinkle, Eva‐Maria Stoegerer, Fernando Rivadeneira, Florian Ernst, Gabriel P. Krestin, Georg Homuth, Gerardo Heiss, Gianluca Usala, Gonçalo R. Abecasis, Gudny Eiriksdottir, Haiqing Shen, H. Erich Wichmann, Helena Schmidt, Henry Völzke, Ingrid B. Borecki, Hugh S. Markus, Jacqueline Witteman, James F. Wilson, Jan Lüdemann, Jeffrey R. O'Connell, Jennifer E. Huffman, Jens Baumert, Jerome I. Rotter, Joanne M. Murabito, Joachim Thiery, Jochen Seissler, Jorma Viikari, Joseph M. Massaro, Joseph F. Polak, Julie Cunningham, Joshua C. Bis, Kari North, Katja E. Petrovic, Kenneth Rice, Kent Taylor, L. Adrienne Cupples, Lawrence F. Bielak, Leena Peltonen, Lenore J. Launer, Mariza de Andrade, Manuela Uda, Marco Orru, Marcus Dörr, Mary F. Feitosa, Maryam Kavousi, Matthias Sitzer, Matthijs Oudkerk, Michael A. Province, Michael Nalls, Mika Kähönen, Muredach P. Reilly, Nicole L. Glazer, Nora Franceschini, Norman Klopp, Olli Raitakari, Patricia A. Peyser, Philip A. Wolf, Qunyuan Zhang, Philipp S. Wild, Renate B. Schnabel, Roberto Elosua, Ralph B. D'Agostino, Ravi Kumar Chilukoti, Reinhold Schmidt, Renate B. Schnabel, Sekar Kathiresan, Serena Sanna, Sharon L. R. Kardia, Shih‐Jen Hwang, Serkalem Demissie, Sigurdur Sigurdsson, Stephen M. Schwartz, Stefan Blankenberg, Steve Bevan, Suzette E. Elias‐Smale, Susan R. Heckbert, Tamara B. Harris, Tanja Zeller, Terho Lehtimäki, Thomas Illig, Thomas Münzel, Thor Aspelund, Timothy D. Howard, Udo Hoffmann, Ulf Schminke, Veikko Salomaa, Vijay Nambi, Vilmundur Gudnason, Yongmei Liu, Yan V. Sun, Wendy S. Post, Wolfgang Koenig, Wolfgang Rathmann, Xia Li, Yu‐Ching Cheng

**Affiliations:** ^1^ Framingham Heart Study Framingham MA; ^2^ The Population Sciences Branch Division of Intramural Research National Heart, Lung, and Blood Institute Bethesda MD; ^3^ Department of Public Health and Primary Care University of Cambridge United Kingdom; ^4^ Cardiology Section and Center for Population Genomics Boston Veteran's Administration (VA) Healthcare Boston MA

**Keywords:** coronary heart disease, genome‐wide association study, Mendelian randomization, plasminogen activator inhibitor type 1, single nucleotide polymorphism, Genetic, Association Studies, Epidemiology, Risk Factors, Coronary Artery Disease

## Abstract

**Background:**

Plasminogen activator inhibitor type 1 (PAI‐1) plays an essential role in the fibrinolysis system and thrombosis. Population studies have reported that blood PAI‐1 levels are associated with increased risk of coronary heart disease (CHD). However, it is unclear whether the association reflects a causal influence of PAI‐1 on CHD risk.

**Methods and Results:**

To evaluate the association between PAI‐1 and CHD, we applied a 3‐step strategy. First, we investigated the observational association between PAI‐1 and CHD incidence using a systematic review based on a literature search for PAI‐1 and CHD studies. Second, we explored the causal association between PAI‐1 and CHD using a Mendelian randomization approach using summary statistics from large genome‐wide association studies. Finally, we explored the causal effect of PAI‐1 on cardiovascular risk factors including metabolic and subclinical atherosclerosis measures. In the systematic meta‐analysis, the highest quantile of blood PAI‐1 level was associated with higher CHD risk comparing with the lowest quantile (odds ratio=2.17; 95% CI: 1.53, 3.07) in an age‐ and sex‐adjusted model. The effect size was reduced in studies using a multivariable‐adjusted model (odds ratio=1.46; 95% CI: 1.13, 1.88). The Mendelian randomization analyses suggested a causal effect of increased PAI‐1 level on CHD risk (odds ratio=1.22 per unit increase of log‐transformed PAI‐1; 95% CI: 1.01, 1.47). In addition, we also detected a causal effect of PAI‐1 on elevating blood glucose and high‐density lipoprotein cholesterol.

**Conclusions:**

Our study indicates a causal effect of elevated PAI‐1 level on CHD risk, which may be mediated by glucose dysfunction.

## Introduction

Plasminogen activator inhibitor type 1 (PAI‐1) is the major inhibitor of the fibrinolytic system. It inhibits the effect of plasminogen activators, thereby inhibiting plasmin formation and downregulating breakdown of fibrin clots. PAI‐1 deficiency caused by mutations has been reported to lead to a moderate bleeding disorder.^1^ On the flip side, high levels of PAI‐1 were reported in some families with thrombophilia.^2^, ^3^ Blood PAI‐1 antigen levels and activity are strongly correlated and both measures have been used to study the role of PAI‐1 in cardiovascular disease. Elevated PAI‐1 levels have been observed to be associated with reinfarction and coronary heart disease (CHD) generally.^4^ Furthermore, circumventing PAI‐1 actions by administering tissue plasminogen activator, one of the key targets inhibited by PAI‐1, is an important treatment for acute ischemic stroke.^5^

However, the relationship between PAI‐1 and early atherosclerosis and incident CHD remains unclear. Previous studies reported the correlation of PAI‐1 with multiple conventional risk factors of CHD, eg, obesity, glycemic traits, and type 2 diabetes mellitus,^6^ metabolic syndrome,^7^ as well as correlation with vessel wall thickness.^8^ In addition, higher PAI‐1 expression was observed in coronary artery tissues in the presence of atherogenic lesions.^9^, ^10^ These findings raise interest in whether PAI‐1 plays a role in early atherosclerosis versus acute thrombosis. The association of elevated plasma PAI‐1 levels with CHD incidence has been reported in longitudinal studies.^11^, ^12^, ^13^, ^14^, ^15^, ^16^, ^17^, ^18^, ^19^ However, this association did not always remain consistent after adjusting for cardiovascular risk factors.^11^, ^12^, ^13^, ^14^, ^16^, ^17^, ^18^, ^19^, ^20^, ^21^, ^22^, ^23^ On the one hand, these inconsistencies could be due to small sample sizes and/or restricted study populations (eg, type 2 diabetes mellitus patients, obese individuals, or HIV patients).^20^, ^24^ On the other hand, the original observational associations including the link between PAI‐1 and CHD are potentially prone to bias from unmeasured confounders or overadjustment for mediators.

To overcome these obstacles, epidemiological studies have adapted instrumental variable (IV) analysis to assess causality and to limit confounding through the use of single nucleotide polymorphisms (SNPs) as IV. This method is referred to as the Mendelian randomization (MR) approach.^25^ Given that genotypes are assigned randomly from parents to offspring during meiosis, the causal effect of PAI‐1 on CHD risk can be estimated by the ratio of a SNP(IV)‐PAI‐1 association to SNP(IV)‐CHD association.^25^, ^26^ Using the MR method, a previous study suggested a causal association of PAI‐1 with myocardial infarction and blood triglycerides.^27^ However, in that study, the association of the SNP, the “4G/5G” polymorphism (rs1799889) in *SERPINE1,* with PAI‐1 and CHD risk was based on a meta‐analysis using published candidate gene studies between 1993 and 2010.^27^ Those observations could be influenced by publication bias, small sample sizes and testing of a single SNP IV. With the recent advent of large‐scale genetic studies, an updated view of potential causal associations between PAI‐1, CHD, and its risk factors is warranted, and should have better power to untangle potential causal pathways.

In the largest genome‐wide association study (GWAS) for PAI‐1 (n=19 599 individuals of European ancestry), the CHARGE Hemostatic Working Group reported 4 independent genetic variants from 3 loci (chr7q22.1, chr11p15.2, chr3p25.2).^28^ The strongest finding in the study was the *SERPINE1* gene locus, the coding gene region of PAI‐1, on chr7q22.1. The lead SNP rs2227631 is in the promoter region of *SERPINE1* and highly correlated with the well‐characterized functional variant 4G/5G *SERPINE1* polymorphism (*r* ^2^=0.78). Following conditional analysis for the lead SNP, a second independent signal (rs6976053) in the same chr7q22.1 locus was observed 200 kb upstream of rs2227631.^28^ In total, the genetic variants from the 3 identified loci explained 0.9% variation of plasma PAI‐1 levels in the Framingham Heart Study.^28^ This strength of IV is in the range of others that have been employed in successful MR studies, suggesting these variants could serve as a potential IV in MR analyses with risk factor, subclinical, and clinical outcomes.^26^, ^29^, ^30^

In this investigation, we aimed to understand whether plasma PAI‐1 levels played a causal role in CHD risk. To achieve the goal, we first investigated the observational association between PAI‐1 and CHD using a systematic meta‐analysis. We then explored the causal effect of PAI‐1 on CHD using a MR approach. Finally, we further investigated the causal effect of PAI‐1 on known cardiovascular risk factors, including metabolic risk factors (ie, type 2 diabetes mellitus, body mass index [BMI], waist‐hip ratio, fasting blood glucose, insulin and lipids, and blood pressure) and subclinical atherosclerosis measures (ie, carotid intima‐media thickness, carotid plaque volume, and coronary artery calcification).

## Methods

### Systematic Meta‐Analysis for Observational Association

**Figure 1:**
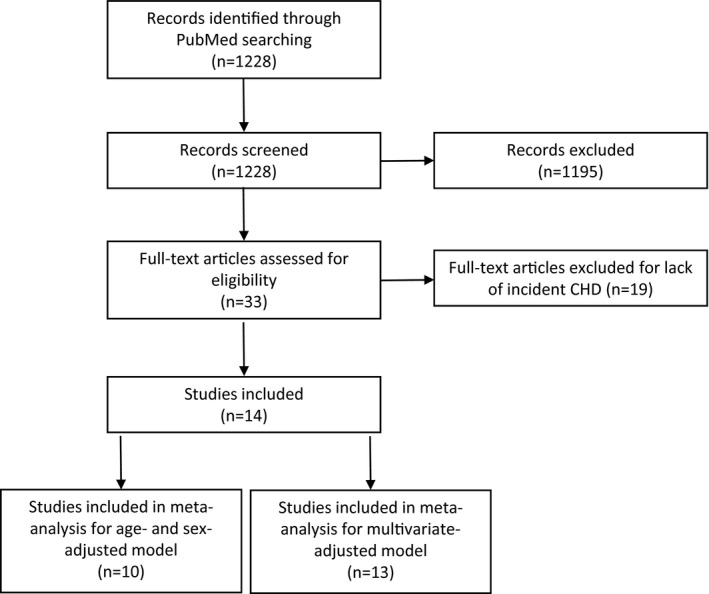
Flow chart for article selection in systematic meta‐analysis. CHD indicates coronary heart disease.

We applied a systematic review to understand the observational association of PAI‐1 with CHD. An electronic literature search was conducted in PubMed by 2 researchers independently using the following criteria: (1) including “Coronary heart disease” or “Coronary artery disease” or “Myocardial infarction”; (2) including “plasminogen activator inhibitor type 1”; (3) published in English from January 1992 to April 2016; and (4) study in human subjects. Two reviewers independently performed the literature screen and found consistent results. In total, we found 1228 articles available in PubMed. There were 33 publications that reported an effect size of PAI‐1 on CHD. To focus on association between PAI‐1 and incident CHD, we excluded those studies that reported prevalent CHD (13 publications), recurrent CHD (5 publications), or stroke (1 publication). In addition, considering that the majority of publications reported the relative risk of CHD comparing the highest (ie, tertile, quartile, or quintile) with the lowest quantile, we included only publications using categorical analyses of PAI‐1. Ten studies that adjusted only for age and sex reported association between PAI‐1 and CHD incidence (Table S1), while 13 studies found the same association following adjustment for multiple covariates (eg, BMI, blood glucose, blood lipids, and blood pressure; Table S1).^11^, ^12^, ^13^, ^14^, ^15^, ^16^, ^17^, ^18^, ^19^, ^20^, ^21^, ^22^, ^23^, ^31^ Covariates used in each study that included multivariable‐adjusted models are shown in Table S1. Detailed literature screening procedure is presented in Figure 1. A random‐effect meta‐analysis was applied in each group of studies using the “metan” package in STATA 13.1.

### Instrumental Variable Analysis for Causal Association

A genetic variant acts as an IV if it fulfils the following assumptions: (1) the genetic variant is associated with the exposure; and (2) the genetic variant can only influence the outcome through the exposure.^32^ Burgess et al reported that MR can be applied for causal association using summary genetic statistics, ie, beta coefficients with standard errors from genetic association studies.^25^ An MR approach using summary data can take advantage of the statistical power of large sample sizes of previous GWASs and does not require multiple phenotypes to be measured in the same study sample. We conducted a power calculation (https://sb452.shinyapps.io/power/) for a PAI‐1 IV (explaining 0.9% of variance).^28^ The results suggest we have 80% statistical power to find a causal odds ratio (OR) larger than 1.15. Thus, we considered the MR analyses viable and obtained summary statistics for circulating PAI‐1 levels, CHD and CHD risk factors from previous GWASs as listed in Table 1. These are based on the largest GWAS meta‐analysis for each phenotype at the time of analysis and primarily conducted on European ancestry samples.^28^, ^33^, ^34^, ^35^, ^36^, ^37^, ^38^, ^39^, ^40^, ^41^, ^42^

**Table 1:** List of Genome‐Wide Association Studies Used in the Current Study

Trait	Consortium	Sample Sizes	Unit	Transformation	Reference
SNP association with the exposure					
PAI‐1	CHARGE	19 599	ng/mL	log‐transformed	Huang et al^28^
SNP association with the primary outcome					
CHD^a^	CARDIOGRAMplusC4D	60 801/123 504^b^	Case/control	N/A	Nikpay et al^39^
SNP association with the potential intermediators via metabolic syndrome					
Type 2 diabetes mellitus	DIAGRAM	34 840/114 981^b^	Case/control	N/A	Morris et al^38^
Fasting blood glucose	MAGIC	58 074	mmol/L	N/A	Manning et al^37^
Fasting blood insulin	MAGIC	51 750	pmol/L	Log‐transformed	Manning et al^37^
Blood lipids^c^	GLGC	188 577	mmol/L	Quantile normalization	Willer et al^42^
Blood pressure^d^	ICBP	203 056	mm Hg	Inverse standard normalization	Ehret et al^35^
BMI	GIANT	339 224	kg/m^2^	Inverse standard normalization	Locke et al^36^
Waist–hip ratio	GIANT	224 459	cm/cm	Inverse standard normalization	Shungin et al^41^
Adiponectin	ADIPOGen	39 883	μg/mL	Log‐transformed	Dastani et al^34^
SNP association with the potential intermediators via early atherosclerosis					
IMT	CHARGE	31 211	mm	Log‐transformed	Bis et al^33^
Carotid plaque^e^	CHARGE	12 955/18 263^b^	Case/control	N/A	Bis et al^33^
CAC	CHARGE	9961	Agatston score	Log‐transformed	O'Donnell et al^40^

BMI indicates body mass index; CAC, coronary artery calcification; CHD, coronary heart disease; IMT, intima‐media thickness; N/A, not applicable; PAI‐1, plasminogen activator type 1; SNP, single nucleotide polymorphism.

a CHD cases include myocardial infarction and unstable angina.

b Sample sizes of CHD, type 2 diabetes mellitus, and plaque are split into cases and controls.

c Blood lipids includes serum total cholesterol, high‐density lipoprotein cholesterol, low‐density lipoprotein cholesterol, and triglycerides.

d Blood pressure includes systolic blood pressure and diastolic blood pressure measures.

e Plaque cases are individuals with presence of carotid plaque.

We applied 2 sets of genetic variants as IVs. First, we selected multiple genetic variants from the PAI‐1 locus chr7q22.1 (*SERPINE1*). In this step, we selected SNPs in this locus that were associated with PAI‐1 (*P*<1×10^−6^) and that were only moderately correlated with each other after iterative stepwise selection (*r* ^2^<0.5 each round). The correlations between SNPs were obtained from the bioinformatics tool SNiPA using data from the 1000 Genomes phase 3, European reference population.^43^ This resulted in 4 selected SNPs (rs2227631, rs2075756, rs12672665, and rs757718; Table S2). A genetic risk score as IV was then constructed by adding the number of risk alleles and weighting each risk allele dose by its effect on PAI‐1. We further corrected for the correlation between each SNP in this genetic risk score in our IV analysis following the method developed by Burgess et al.^44^ In the second step, we extended the genetic risk score by using multiple loci from the PAI‐1 GWAS that were shown to reach genome‐wide significance (*P*<5×10^−8^). Four independent SNPs reported from the PAI‐1 GWAS were used in the IV (rs2227631, rs6976053, rs6486122, and rs11128603; Table S2),^28^ and then a genetic risk score was generated as IV by adding the counts of risk alleles weighted by their effects on PAI‐1. The second IV was only applied for the causal association between PAI‐1 and CHD, but not between PAI‐1 and cardiovascular risk factors.

The estimates for associations of SNPs with PAI‐1 are reported per risk allele change of units of log‐transformed PAI‐1.^28^ The estimates for associations of SNPs with CHD are reported per risk allele change of CHD risk.^39^ The causal effect of a per unit change of log‐transformed PAI‐1 on CHD is estimated as the per risk allele change of CHD risk dependent on the per risk allele change of log‐transformed PAI‐1 units. When using 1 SNP as an IV, it is calculated as the ratio estimate of the SNP‐CHD association to the SNP‐PAI‐1, that is, β_SNP‐CHD association_/β_SNP‐PAI‐1 association_.^26^ When using multiple SNPs as an IV, the combined causal effect was evaluated as the inverse‐variance weighted estimate of the causal ratio when using each SNP alone as an IV.^25^ The 95% CIs for causal estimates are calculated based on the estimates (beta/log‐transformed OR) and SE: estimate±1.96×SE. The IV analysis was performed using R version 3.1.2.

## Results

**Figure 2:**
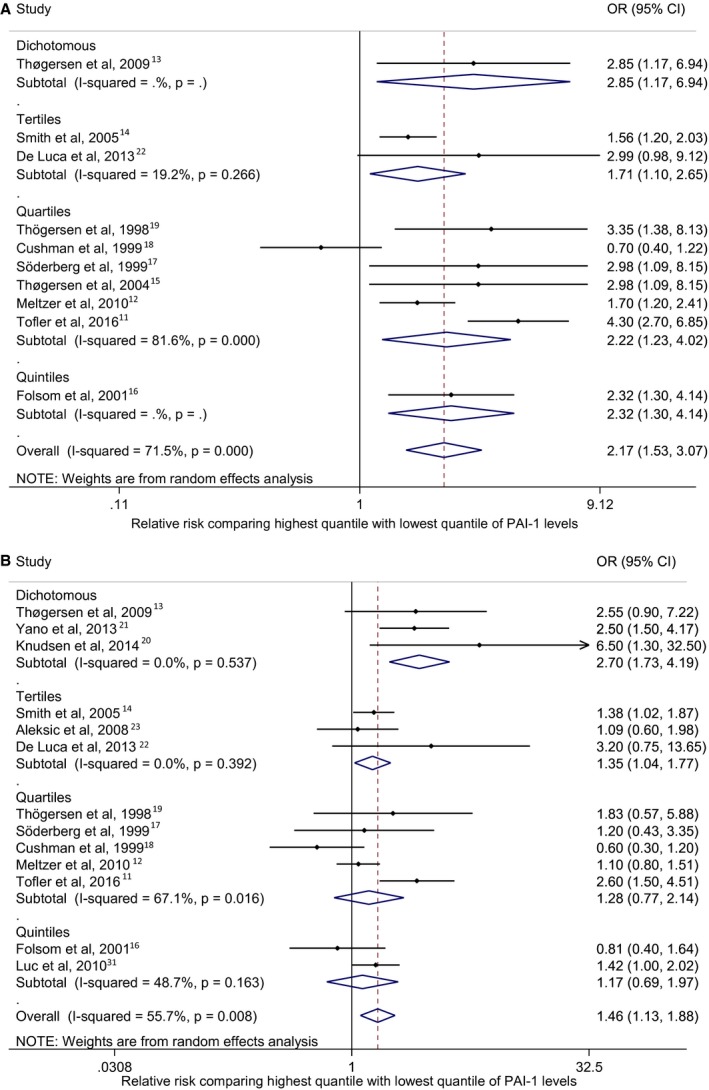
Observational associations of PAI‐1 and CHD from the literature up until July 2016. This is a forest plot depicting the result of the meta‐analysis based on previous publications for observations of plasminogen activator inhibitor type 1 (PAI‐1) and coronary heart disease (CHD) association. Odds ratio (OR) with 95% CI is the OR of CHD comparing the highest PAI‐1 quantile to lowest PAI‐1 quantile, which is also represented as a point with bar in the plot. The diamond represents the meta‐analysis result using a random effects model. A, PAI‐1‐CHD associations adjusted for age, sex, and ethnic group in each study. B, PAI‐1‐CHD association in models adjusted for multiple CHD risk factors.

In the systematic review, most previous studies reported PAI‐1 and CHD incidence association based on PAI‐1 levels in quartiles, while there were 3 studies based on dichotomizing PAI‐1 levels, 3 using tertiles and 2 using quintiles. A pooled meta‐analysis shows the highest quantile (ie, tertile, quartile, or quintile) of blood PAI‐1 levels is associated with higher risk of CHD incidence compared with the lowest quantile (OR=2.17; 95% CI: 1.53, 3.07; Figure 2A) in an age‐and sex‐adjusted model.^11^, ^12^, ^13^, ^14^, ^15^, ^16^, ^17^, ^18^, ^19^, ^22^ The overall heterogeneity across studies is high (I^2^=71.5%, *P*<0.001; Figure 2A). The association estimate is reduced but remains significant in studies using a multivariable‐adjusted model (OR=1.46; 95% CI: 1.13, 1.88; Figure 2B),^11^, ^12^, ^13^, ^14^, ^16^, ^17^, ^18^, ^19^, ^20^, ^21^, ^22^, ^23^, ^31^ with a lower overall heterogeneity compared with studies applying only an age‐and sex‐adjusted model (I^2^=55.7%, *P*=0.008; Figure 2B). In the subgroup meta‐analysis based on different PAI‐1 quantile scales, heterogeneity is observed most strongly in the quartile subgroup in both age‐ and sex‐adjusted model (I^2^=81.6%, *P*<0.001; Figure 2A) and the multivariable‐adjusted model (I^2^=61.7%, *P*<0.016; Figure 2B).

**Figure 3:**
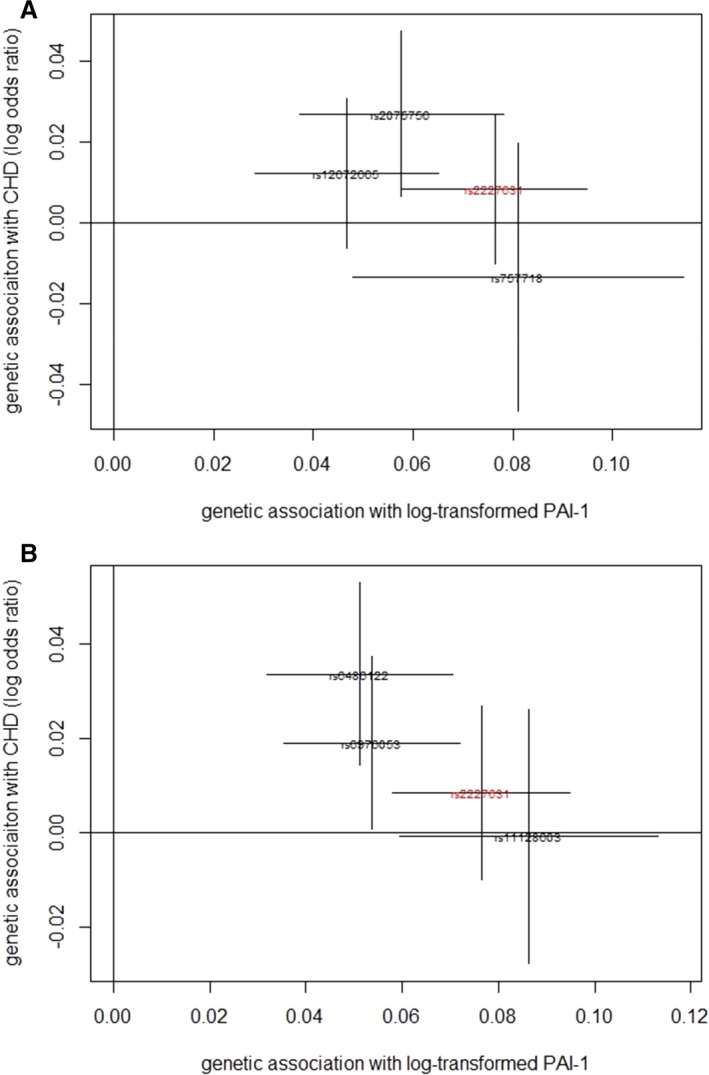
Scatter plots for genetic associations with CHD against genetic associations with PAI‐1. This shows scatter plots of genetic associations with coronary heart disease (CHD) against genetic associations with plasminogen activator inhibitor type 1 (PAI‐1). Lines in the horizontal and vertical directions represent 95% CI of genetic associations with PAI‐1 and CHD, respectively. The SNP marked in red (rs2227631) located in *SERPINE1* is the SNP with the lowest *P*‐value reported in the genome‐wide association study of PAI‐1.^28^ A, Scatter plot of 4 correlated SNPs selected in the *SERPINE1* locus as instrumental variable. B, Scatter plot of 4 independent SNPs selected from multiple loci as instrumental variable. SNP indicates single nucleotide polymorphism.

Using variants in the *SERPINE1* locus as IVs, the MR analysis suggests that, under the assumptions of the MR approach, an increase of one unit of log‐transformed PAI‐1 level can increase CHD risk by 22% (OR=1.22; 95% CI: 1.02, 1.45; Table 2). When variants in multiple loci are used as IVs, the result is consistent and the confidence interval narrows slightly (OR=1.25; 95% CI: 1.07, 1.45). We constructed scatter plots for genetic associations with CHD against genetic associations with PAI‐1 for the 2 sets of SNPs as IV separately (Figure 3). Figure 3A shows that all SNPs from the *SERPINE1* locus are associated with PAI‐1 and CHD in a similar manner. That is, when one SNP has a larger effect size on PAI‐1, it also has a relatively larger effect size on CHD risk. This suggests that the results using the combined genetic risk score in set 1 (*SERPINE1* locus) as IV are not simply driven by a single SNP. Similarly, Figure 3B suggests that the results when using multiple loci in the IV are not driven by a single SNP.

**Table 2:** Causal Effect of PAI‐1 on Cardiovascular Risk Factors Using the *SERPINE1* Locus as IV

Trait	Effect	95% CI	*P* Value
CHD^a^	1.22	(1.01, 1.47)	0.039
Metabolic risk factors			
Type 2 diabetes mellitus^a^	1.18	(0.85, 1.62)	0.321
Fasting blood glucose	0.08	(0.02, 0.14)	0.012
Fasting blood insulin	−0.002	(−0.07, 0.06)	0.939
Total cholesterol	0.08	(−0.02, 0.19)	0.113
HDL‐C	0.13	(0.04, 0.23)	0.008
LDL‐C	0.03	(−0.08, 0.13)	0.583
Triglycerides	−0.03	(−0.12, 0.23)	0.578
BMI	−0.07	(−0.14, 0.01)	0.070
Waist–hip ratio	−0.07	(−0.15, 0.02)	0.112
Systolic blood pressure	1.22	(−0.77, 3.20)	0.230
Diastolic blood pressure	0.20	(−1.06, 1.46)	0.758
Adiponectin	0.004	(−0.08, 0.09)	0.926
Subclinical atherosclerosis			
IMT	0.01	(−0.02, 0.04)	0.669
Carotid plaque^a^	1.03	(0.98, 1.58)	0.876
CAC	0.33	(−0.29, 0.95)	0.293

BMI indicates body mass index; CAC, coronary artery calcification; CHD, coronary heart disease; HDL‐C, high‐density lipoprotein cholesterol; IMT, intima‐media thickness; IV, instrumental variable; LDL‐C, low‐density lipoprotein cholesterol; PAI‐1, plasminogen activator type 1.

a The traits with marked with * are dichotomous traits and effects/95% CI for these traits were reported as odds ratio. Other traits are all continuous traits, and their effects/95% CI were reported as beta coefficients.

MR analyses for causal effects of PAI‐1 on cardiovascular risk factors using variants in the *SERPINE1* locus alone suggest that an increase of 1 unit of log‐transformed PAI‐1 level increases circulating fasting glucose levels by 0.08 mmol/L (β=0.08; 95% CI: 0.02, 0.14; Table 2); and increases high‐density lipoprotein cholesterol (HDL‐C) by 0.13 SDs (β=0.13; 95% CI: 0.04, 0.23; Table 2). We found no evidence for causal effects of PAI‐1 on other metabolic risk factors, or subclinical atherosclerosis. However, the result for BMI suggests a negative effect of PAI‐1 on BMI with a trend toward significance (β=−0.07, *P*=0.070; Table 2), which contradicts most epidemiological studies demonstrating a positive association.^45^, ^46^ Therefore, we further investigated the causal effect of BMI on PAI‐1 levels using 77 genome‐wide significant loci identified in a large BMI GWAS in European populations (Data S1).^36^ This result shows that BMI has a causal effect on PAI‐1 in the positive direction (β: 0.21; 95% CI: 0.13, 0.29; Table S3), and was consistent with sensitivity analyses using a median estimator approach and MR‐Egger regression to test for potential pleiotropic effects.

## Discussion

The systematic meta‐analysis using the available epidemiological literature supports the association between PAI‐1 and CHD incidence, independent of established cardiovascular risk factors. Given the heterogeneity across studies, we further utilized the MR approach. This approach has been successful in supporting (low‐density lipoprotein cholesterol) and refuting (HDL‐C) causal links to CHD that mirror clinical trial results.^47^, ^48^ The results of our MR study do support a causal link between PAI‐1 and CHD. In addition, the MR analyses also suggest a casual effect of PAI‐1 levels on blood glucose levels and HDL‐C levels. Our study represents a comprehensive investigation of the effect of PAI‐1 on CHD and its risk factors in well‐powered population samples, suggesting potential mechanisms for further investigation or potential intervention. We investigated the association between PAI‐1 and incident CHD via systematic meta‐analysis using current publications. Additionally, this study is the first to report the causality of PAI‐1 on CHD and CHD risk factors using GWAS summary statistics. By leveraging large sample sizes reaching over 60 000 cases and 120 000 controls from GWAS consortia, we find a robust causal association of PAI‐1 with CHD.

A key assumption for the MR approach is that genetic variants employed as the IV can only be associated with the outcome (CHD) through the biomarker (PAI‐1). The causal effect of PAI‐1 on CHD suggested by the MR approach should be interpreted under this assumption. In addition, further functional studies are required to understand the mechanism of the causal association between PAI‐1 and CHD. As a protein biomarker, the genetic locus encoding the PAI‐1 transcript has clear biological function in determining circulating PAI‐1 levels. The 4G/5G polymorphism in the promoter region of *SERPINE1* has been consistently reported to be a functional variant influencing PAI‐1 expression.^27^, ^49^ Knockout of *Serpine1,* a mouse ortholog, creates PAI‐1 deficiency.^50^ Therefore, when exploring whether metabolic risk factors and subclinical atherosclerosis are mediators of potential PAI‐1 effects on CHD, we only used the *SERPINE1* locus SNPs as an IV.

Our study is the first evidence to suggest a causal association of PAI‐1 on increased fasting glucose. This indicates PAI‐1 may play a role in glucose regulation and is consistent with previous population studies that reported positive correlations between circulating PAI‐1 and glucose levels.^6^, ^51^, ^52^ In addition to observational studies, experimental studies showed that PAI‐1 deficiency via genetic knock‐out or pharmacological inhibition can suppress the levels of blood glucose in mice.^53^, ^54^ MR analysis of PAI‐1 with type 2 diabetes mellitus had a consistent effect direction with what is expected based on the glucose findings, but was not significant (Table 2). This finding potentially suggests a causal pathway of PAI‐1 to CHD risk, mediated by elevated glucose level. However, a mediation test would be required to verify this conclusion using individual‐level data with genetics, PAI‐1 levels, fasting glucose, and CHD in the same study population.

Somewhat surprisingly, when addressing the causal effect of PAI‐1 on measurements of obesity, we find negative trend effects of PAI‐1 on BMI and waist–hip ratio. Adipose tissue is one of the main tissues expressing PAI‐1, and population studies have consistently shown positive correlations between circulating PAI‐1 levels and BMI.^51^ Ex vivo studies suggested the bidirectional regulation between PAI‐1 and adipocytes. For example, Crandall et al suggested that endogenous expression of PAI‐1 might regulate adipogenesis by preventing preadipocyte migration into cell clusters.^55^ Halleux et al reported that the expression of PAI‐1 in cultured human adipose tissue elevated in response to glucocorticoids.^56^ Therefore, we conducted analysis on the causal effect of BMI on PAI‐1 levels. The results indicated a potential positive causal effect of BMI on PAI‐1. Taken together, we conclude that the association between circulating PAI‐1 levels and BMI may be due to a causal effect of BMI on PAI‐1 rather than PAI‐1 regulation on BMI.

Our results suggest a further positive causal effect of PAI‐1 on HDL‐C, which is inconsistent with observational associations in the population study.^51^ However, an HDL‐C influence on CHD is itself paradoxical. While observational studies consistently report a protective association of high blood HDL‐C levels with lower CHD risk, previous MR studies show that HDL‐C is not a causal risk factor for CHD.^48^, ^57^ Furthermore, a recent study has reported that a loss‐of‐function variant in scavenger receptor BI (*SRB1*) raises HDL‐C and increases CHD risk.^58^ Further studies are warranted to understand potential biological mechanisms of a PAI‐1 effect on HDL‐C and whether HDL‐C is an intermediary between the PAI‐1 and CHD associations.

There are several limitations of the current study. Since we used summary GWAS statistics in the current study, we were unable to address stratified analysis questions such as whether there is a sex or age difference in the PAI‐1‐CHD link, or whether the effect of PAI‐1 on CHD differs among obese individuals versus nonobese individuals. These are interesting questions for future studies. In addition, our reported observational meta‐analysis between PAI‐1 and CHD is based on PAI‐1 quantiles, while the causal association is based on log‐transformed PAI‐1 units; therefore, the effect size of PAI‐1 on CHD in these 2 sets of analysis is not directly comparable.

In summary, we applied several approaches to understand the role of PAI‐1 in CHD. Our results through several analyses support a causal effect of PAI‐1 on CHD onset, potentially mediated by blood glucose dysfunction. Furthermore, our results and those of others suggest that PAI‐1 may be interlocked with obesity, and potentially HDL‐C in complex feedback relationships. Our study adds to evidence on the role of PAI‐1 in pathogenesis of CHD and suggests this pathway may be a good target for CHD treatment.

## Appendix

### CHARGE Consortium Hemostatic Factor Working Group

Jie Huang, Maria Sabater‐Lleal, Folkert W. Asselbergs, David Tregouet, So‐Youn Shin, Jingzhong Ding, Jens Baumert, Tiphaine Oudot‐Mellakh, Lasse Folkersen, Andrew D. Johnson, Nicholas L. Smith, Scott M. Williams, Mohammad A. Ikram, Marcus E. Kleber, Diane M. Becker, Vinh Truong, Josyf C. Mychaleckyj, Weihong Tang, Qiong Yang, Bengt Sennblad, Jason H. Moore, Frances M. K. Williams, Abbas Dehghan, Günther Silbernagel, Elisabeth M. C. Schrijvers, Shelly Smith, Mahir Karakas, Geoffrey H. Tofler, Angela Silveira, Gerjan J. Navis, Kurt Lohman, Ming‐Huei Chen, Annette Peters, Anuj Goel, Jemma C. Hopewell, John C. Chambers, Danish Saleheen, Per Lundmark, Bruce M. Psaty, Rona J. Strawbridge, Bernhard O. Boehm, Angela M. Carter, Christa Meisinger, John F. Peden, Joshua C. Bis, Barbara McKnight, John Öhrvik, Kent Taylor, Maria Grazia Franzosi, Udo Seedorf, Rory Collins, Anders Franco‐Cereceda, Ann‐Christine Syvänen, Alison H. Goodall, Lisa R. Yanek, Mary Cushman, Martina Müller‐Nurasyid, Aaron R. Folsom, Saonli Basu, Nena Matijevic, Wiek H. van Gilst, Jaspal S. Kooner, Albert Hofman, John Danesh, Robert Clarke, James B. Meigs, Sekar Kathiresan, Muredach P. Reilly, Norman Klopp, Tamara B. Harris, Bernhard R. Winkelmann, Peter J. Grant, Hans L. Hillege, Hugh Watkins, Timothy D. Spector, Lewis C. Becker, Russell P. Tracy, Winfried März, Andre G. Uitterlinden, Per Eriksson, Francois Cambien, Pierre‐Emmanuel Morange, Wolfgang Koenig, Nicole Soranzo, Pim van der Harst, Yongmei Liu, Christopher J. O'Donnell, and Anders Hamsten.

### ICBP Consortium

Georg B. Ehret, Patricia B. Munroe, Kenneth M. Rice, Murielle Bochud, Andrew D. Johnson, Daniel I. Chasman, Albert V. Smith, Martin D. Tobin, Germaine C. Verwoert, Shih‐Jen Hwang, Vasyl Pihur, Peter Vollenweider, Paul F. O'Reilly, Najaf Amin, Jennifer L. Bragg‐Gresham, Alexander Teumer, Nicole L. Glazer, Lenore Launer, Jing Hua Zhao, Yurii Aulchenko, Simon Heath, Siim Sõber, Afshin Parsa, Jian'an Luan, Pankaj Arora, Abbas Dehghan, Feng Zhang, Gavin Lucas, Andrew A. Hicks, Anne U. Jackson, John F. Peden, Toshiko Tanaka, Sarah H. Wild, Igor Rudan, Wilmar Igl, Yuri Milaneschi, Alex N. Parker, Cristiano Fava, John C. Chambers, Ervin R. Fox, Meena Kumari, Min Jin Go, Pim van der Harst, Wen Hong Linda Kao, Marketa Sjögren, D. G. Vinay, Myriam Alexander, Yasuharu Tabara, Sue Shaw‐Hawkins, Peter H. Whincup, Yongmei Liu, Gang Shi, Johanna Kuusisto, Bamidele Tayo, Mark Seielstad, Xueling Sim, Khanh‐Dung Hoang Nguyen, Terho Lehtimäki, Giuseppe Matullo, Ying Wu, Tom R. Gaunt, N. Charlotte Onland‐Moret, Matthew N. Cooper, Carl G. P. Platou, Elin Org, Rebecca Hardy, Santosh Dahgam, Jutta Palmen, Veronique Vitart, Peter S. Braund, Tatiana Kuznetsova, Cuno S. P. M. Uiterwaal, Adebowale Adeyemo, Walter Palmas, Harry Campbell, Barbara Ludwig, Maciej Tomaszewski, Ioanna Tzoulaki, Nicholette D. Palmer, Thor Aspelund, Melissa Garcia, Yen‐Pei C. Chang, Jeffrey R. O'Connell, Nanette I. Steinle, Diederick E. Grobbee, Dan E. Arking, Sharon L. Kardia, Alanna C. Morrison, Dena Hernandez, Samer Najjar, Wendy L. McArdle, David Hadley, Morris J. Brown, John M. Connell, Aroon D. Hingorani, Ian N. M. Day, Debbie A. Lawlor, John P. Beilby, Robert W. Lawrence, Robert Clarke, Jemma C. Hopewell, Halit Ongen, Albert W. Dreisbach, Yali Li, J. Hunter Young, Joshua C. Bis, Mika Kähönen, Jorma Viikari, Linda S. Adair, Nanette R. Lee, Ming‐Huei Chen, Matthias Olden, Cristian Pattaro, Judith A. Hoffman Bolton, Anna Köttgen, Sven Bergmann, Vincent Mooser, Nish Chaturvedi, Timothy M. Frayling, Muhammad Islam, Tazeen H. Jafar, Jeanette Erdmann, Smita R. Kulkarni, Stefan R. Bornstein, Jürgen Grässler, Leif Groop, Benjamin F. Voight, Johannes Kettunen, Philip Howard, Andrew Taylor, Simonetta Guarrera, Fulvio Ricceri, Valur Emilsson, Andrew Plump, Inês Barroso, Kay‐Tee Khaw, Alan B. Weder, Steven C. Hunt, Yan V. Sun, Richard N. Bergman, Francis S. Collins, Lori L. Bonnycastle, Laura J. Scott, Heather M. Stringham, Leena Peltonen, Markus Perola, Erkki Vartiainen, Stefan‐Martin Brand, Jan A. Staessen, Thomas J. Wang, Paul R. Burton, Maria Soler Artigas, Yanbin Dong, Harold Snieder, Xiaoling Wang, Haidong Zhu, Kurt K. Lohman, Megan E. Rudock, Susan R. Heckbert, Nicholas L. Smith, Kerri L. Wiggins, Ayo Doumatey, Daniel Shriner, Gudrun Veldre, Margus Viigimaa, Sanjay Kinra, Dorairaj Prabhakaran, Vikal Tripathy, Carl D. Langefeld, Annika Rosengren, Dag S. Thelle, Anna Maria Corsi, Andrew Singleton, Terrence Forrester, Gina Hilton, Colin A. McKenzie, Tunde Salako, Naoharu Iwai, Yoshikuni Kita, Toshio Ogihara, Takayoshi Ohkubo, Tomonori Okamura, Hirotsugu Ueshima, Satoshi Umemura, Susana Eyheramendy, Thomas Meitinger, H.‐Erich Wichmann, Yoon Shin Cho, Hyung‐Lae Kim, Jong‐Young Lee, James Scott, Joban S. Sehmi, Weihua Zhang, Bo Hedblad, Peter Nilsson, George Davey Smith, Andrew Wong, Narisu Narisu, Alena Stančáková, Leslie J. Raffel, Jie Yao, Sekar Kathiresan, Christopher J. O'Donnell, Stephen M. Schwartz, M. Arfan Ikram, W. T. Longstreth Jr, Thomas H. Mosley, Sudha Seshadri, Nick R. G. Shrine, Louise V. Wain, Mario A. Morken, Amy J. Swift, Jaana Laitinen, Inga Prokopenko, Paavo Zitting, Jackie A. Cooper, Steve E. Humphries, John Danesh, Asif Rasheed, Anuj Goel, Anders Hamsten, Hugh Watkins, Stephan J. L. Bakker, Wiek H. van Gilst, Charles S. Janipalli, K. Radha Mani, Chittaranjan S. Yajnik, Albert Hofman, Francesco U. S. Mattace‐Raso, Ben A. Oostra, Ayse Demirkan, Aaron Isaacs, Fernando Rivadeneira, Edward G. Lakatta, Marco Orru, Angelo Scuteri, Mika Ala‐Korpela, Antti J. Kangas, Leo‐Pekka Lyytikäinen, Pasi Soininen, Taru Tukiainen, Peter Würtz, Rick Twee‐Hee Ong, Marcus Dörr, Heyo K. Kroemer, Uwe Völker, Henry Völzke, Pilar Galan, Serge Hercberg, Mark Lathrop, Diana Zelenika, Panos Deloukas, Massimo Mangino, Tim D. Spector, Guangju Zhai, James F. Meschia, Michael A. Nalls, Pankaj Sharma, Janos Terzic, M. V. Kranthi Kumar, Matthew Denniff, Ewa Zukowska‐Szczechowska, Lynne E. Wagenknecht, F. Gerald R. Fowkes, Fadi J. Charchar, Peter E. H. Schwarz, Caroline Hayward, Xiuqing Guo, Charles Rotimi, Michiel L. Bots, Eva Brand, Nilesh J. Samani, Ozren Polasek, Philippa J. Talmud, Fredrik Nyberg, Diana Kuh, Maris Laan, Kristian Hveem, Lyle J. Palmer, Yvonne T. van der Schouw, Juan P. Casas, Karen L. Mohlke, Paolo Vineis, Olli Raitakari, Santhi K. Ganesh, Tien Y. Wong, E. Shyong Tai, Richard S. Cooper, Markku Laakso, Dabeeru C. Rao, Tamara B. Harris, Richard W. Morris, Anna F. Dominiczak, Mika Kivimaki, Michael G. Marmot, Tetsuro Miki, Danish Saleheen, Giriraj R. Chandak, Josef Coresh, Gerjan Navis, Veikko Salomaa, Bok‐Ghee Han, Xiaofeng Zhu, Jaspal S. Kooner, Olle Melander, Paul M. Ridker, Stefania Bandinelli, Ulf B. Gyllensten, Alan F. Wright, James F. Wilson, Luigi Ferrucci, Martin Farrall, Jaakko Tuomilehto, Peter P. Pramstaller, Roberto Elosua, Nicole Soranzo, Eric J. G. Sijbrands, David Altshuler, Ruth J. F. Loos, Alan R. Shuldiner, Christian Gieger, Pierre Meneton, Andre G. Uitterlinden, Nicholas J. Wareham, Vilmundur Gudnason, Jerome I. Rotter, Rainer Rettig, Manuela Uda, David P. Strachan, Jacqueline C. M. Witteman, Anna‐Liisa Hartikainen, Jacques S. Beckmann, Eric Boerwinkle, Ramachandran S. Vasan, Michael Boehnke, Martin G. Larson, Marjo‐Riitta Järvelin, Bruce M. Psaty, Gonçalo R. Abecasis, Aravinda Chakravarti, Paul Elliott, Cornelia M. van Duijn, Christopher Newton‐Cheh, Daniel Levy, Mark J. Caulfield and Toby Johnson.

### CHARGE Consortium Subclinical Working Group

Aad van der Lugt, Aaron Isaacs, Abbas Dehghan, Afshin Parsa, Alan R. Shuldiner, Albert Hofman, Albert V. Smith, Aldi T. Kraja, Andre G. Uitterlinden, Andre Uitterlinden, Andreas Ziegler, Andrew D. Johnson, Angelo Scuteri, Anne B. Newman, Arne Schillert, Benjamin F. Voight, Ben Oostra, Bolli Thorsson, Braxton D. Mitchell, Bruce M. Psaty, Caroline Hayward, Caroline S. Fox, Charles C. White, Christa Meisinger, Christie Ballantyne, Christopher J. O'Donnell, Cornelia van Duijn, David Altshuler, David M. Herrington, Daniel H. O'Leary, David S. Siscovick, David J. Couper, Edward G. Lakatta, Eran Halperin, Eric Boerwinkle, Eva‐Maria Stoegerer, Fernando Rivadeneira, Florian Ernst, Gabriel P. Krestin, Georg Homuth, Gerardo Heiss, Gianluca Usala, Gonçalo R. Abecasis, Gudny Eiriksdottir, Haiqing Shen, H. Erich Wichmann, Helena Schmidt, Henry Völzke, Ingrid B. Borecki, Hugh S. Markus, Jacqueline Witteman, James F. Wilson, Jan Lüdemann, Jeffrey R. O'Connell, Jennifer E. Huffman, Jens Baumert, Jerome I. Rotter, Joanne M. Murabito, Joachim Thiery, Jochen Seissler, Jorma Viikari, Joseph M. Massaro, Joseph F. Polak, Julie Cunningham, Joshua C. Bis, Kari North, Katja E. Petrovic, Kenneth Rice, Kent Taylor, L. Adrienne Cupples, Lawrence F. Bielak, Leena Peltonen, Lenore J. Launer, Mariza de Andrade, Manuela Uda, Marco Orru, Marcus Dörr, Mary F. Feitosa, Maryam Kavousi, Matthias Sitzer, Matthijs Oudkerk, Michael A. Province, Michael Nalls, Mika Kähönen, Muredach P. Reilly, Nicole L. Glazer, Nora Franceschini, Norman Klopp, Olli Raitakari, Patricia A. Peyser, Philip A. Wolf, Qunyuan Zhang, Philipp S. Wild, Renate B. Schnabel, Roberto Elosua, Ralph B. D'Agostino Sr, Ravi Kumar Chilukoti, Reinhold Schmidt, Renate B. Schnabel, Sekar Kathiresan, Serena Sanna, Sharon L. R. Kardia, Shih‐Jen Hwang, Serkalem Demissie, Sigurdur Sigurdsson, Stephen M. Schwartz, Stefan Blankenberg, Steve Bevan, Suzette E. Elias‐Smale, Susan R. Heckbert, Tamara B. Harris, Tanja Zeller, Terho Lehtimäki, Thomas Illig, Thomas Münzel, Thor Aspelund, Timothy D. Howard, Udo Hoffmann, Ulf Schminke, Veikko Salomaa, Vijay Nambi, Vilmundur Gudnason, Yongmei Liu, Yan V. Sun, Wendy S. Post, Wolfgang Koenig, Wolfgang Rathmann, Xia Li and Yu‐Ching Cheng.

## Sources of Funding

This work was supported by NHLBI Intramural funds to O'Donnell and Johnson. Stephen Burgess is supported by a fellowship from the Wellcome Trust (100114).

## Disclosures

None.

## Supporting information


**Data S1.**
**Table S1.** Publications Included in the Observational Meta‐Analysis
**Table S2.** SNPs Involved in the Genetic Risk Scores as Instrumental Variable for PAI‐1
**Table S3.** Causal Effect of BMI on PAI‐1

## References

[1] Fay WP , Parker AC , Condrey LR , Shapiro AD . Human plasminogen activator inhibitor‐1 (PAI‐1) deficiency: characterization of a large kindred with a null mutation in the PAI‐1 gene. Blood. 1997;90:204–208. 9207454

[2] Patrassi GM , Sartori MT , Saggiorato G , Boeri G , Girolami A . Familial thrombophila associated with high levels of plasminogen activator inhibitor. Fibrinolysis. 1992;6:99–103.

[3] Nilsson IM , Ljungner H , Tengborn L . Two different mechanisms in patients with venous thrombosis and defective fibrinolysis: low concentration of plasminogen activator or increased concentration of plasminogen activator inhibitor. BMJ. 1985;290:1453–1456. 3922531 10.1136/bmj.290.6480.1453 PMC1415704

[4] Hamsten A , de Faire U , Walldius G , Dahlen G , Szamosi A , Landou C , Blomback M , Wiman B . Plasminogen activator inhibitor in plasma: risk factor for recurrent myocardial infarction. Lancet. 1987;2:3–9. 2885513 10.1016/s0140-6736(87)93050-9

[5] Tissue plasminogen activator for acute ischemic stroke. The National Institute of Neurological Disorders and Stroke rt‐PA Stroke Study Group. N Engl J Med. 1995;333:1581–1587. 7477192 10.1056/NEJM199512143332401

[6] Yarmolinsky J , Bordin Barbieri N , Weinmann T , Ziegelmann PK , Duncan BB , Ines Schmidt M . Plasminogen activator inhibitor‐1 and type 2 diabetes: a systematic review and meta‐analysis of observational studies. Sci Rep. 2016;6:17714. 26813008 10.1038/srep17714 PMC4728395

[7] Smits MM , Woudstra P , Utzschneider KM , Tong J , Gerchman F , Faulenbach M , Carr DB , Aston‐Mourney K , Chait A , Knopp RH , Meigs JB , Boyko EJ , Kahn SE . Adipocytokines as features of the metabolic syndrome determined using confirmatory factor analysis. Ann Epidemiol. 2013;23:415–421. 23535025 10.1016/j.annepidem.2013.03.001 PMC3778191

[8] Salomaa V , Stinson V , Kark JD , Folsom AR , Davis CE , Wu KK . Association of fibrinolytic parameters with early atherosclerosis. The ARIC Study. Atherosclerosis Risk in Communities Study. Circulation. 1995;91:284–290. 7805229 10.1161/01.cir.91.2.284

[9] Lupu F , Bergonzelli GE , Heim DA , Cousin E , Genton CY , Bachmann F , Kruithof EK . Localization and production of plasminogen activator inhibitor‐1 in human healthy and atherosclerotic arteries. Arterioscler Thromb. 1993;13:1090–1100. 7686395 10.1161/01.atv.13.7.1090

[10] Schneiderman J , Sawdey MS , Keeton MR , Bordin GM , Bernstein EF , Dilley RB , Loskutoff DJ . Increased type 1 plasminogen activator inhibitor gene expression in atherosclerotic human arteries. Proc Natl Acad Sci USA. 1992;89:6998–7002. 1495992 10.1073/pnas.89.15.6998 PMC49632

[11] Tofler GH , Massaro J , O'Donnell CJ , Wilson PW , Vasan RS , Sutherland PA , Meigs JB , Levy D , D'Agostino RB Sr . Plasminogen activator inhibitor and the risk of cardiovascular disease: the Framingham Heart Study. Thromb Res. 2016;140:30–35. 26896607 10.1016/j.thromres.2016.02.002 PMC5722217

[12] Meltzer ME , Doggen CJ , de Groot PG , Rosendaal FR , Lisman T . Plasma levels of fibrinolytic proteins and the risk of myocardial infarction in men. Blood. 2010;116:529–536. 20413657 10.1182/blood-2010-01-263103

[13] Thogersen AM , Nilsson TK , Weinehall L , Boman K , Eliasson M , Hallmans G , Jansson JH . Changes in plasma C‐reactive protein and hemostatic factors prior to and after a first myocardial infarction with a median follow‐up time of 8 years. Blood Coagul Fibrinolysis. 2009;20:340–346. 19357504 10.1097/MBC.0b013e32832a5fd1

[14] Smith A , Patterson C , Yarnell J , Rumley A , Ben‐Shlomo Y , Lowe G . Which hemostatic markers add to the predictive value of conventional risk factors for coronary heart disease and ischemic stroke? The Caerphilly Study Circulation. 2005;112:3080–3087. 16286603 10.1161/CIRCULATIONAHA.105.557132

[15] Thogersen AM , Soderberg S , Jansson JH , Dahlen G , Boman K , Nilsson TK , Lindahl B , Weinehall L , Stenlund H , Lundberg V , Johnson O , Ahren B , Hallmans G . Interactions between fibrinolysis, lipoproteins and leptin related to a first myocardial infarction. Eur J Cardiovasc Prev Rehabil. 2004;11:33–40. 15167204 10.1097/01.hjr.0000116824.84388.a2

[16] Folsom AR , Aleksic N , Park E , Salomaa V , Juneja H , Wu KK . Prospective study of fibrinolytic factors and incident coronary heart disease: the Atherosclerosis Risk in Communities (ARIC) Study. Arterioscler Thromb Vasc Biol. 2001;21:611–617. 11304480 10.1161/01.atv.21.4.611

[17] Soderberg S , Ahren B , Jansson JH , Johnson O , Hallmans G , Asplund K , Olsson T . Leptin is associated with increased risk of myocardial infarction. J Intern Med. 1999;246:409–418. 10583712 10.1046/j.1365-2796.1999.00571.x

[18] Cushman M , Lemaitre RN , Kuller LH , Psaty BM , Macy EM , Sharrett AR , Tracy RP . Fibrinolytic activation markers predict myocardial infarction in the elderly. The Cardiovascular Health Study. Arterioscler Thromb Vasc Biol. 1999;19:493–498. 10073948 10.1161/01.atv.19.3.493

[19] Thogersen AM , Jansson JH , Boman K , Nilsson TK , Weinehall L , Huhtasaari F , Hallmans G . High plasminogen activator inhibitor and tissue plasminogen activator levels in plasma precede a first acute myocardial infarction in both men and women: evidence for the fibrinolytic system as an independent primary risk factor. Circulation. 1998;98:2241–2247. 9826309 10.1161/01.cir.98.21.2241

[20] Knudsen A , Katzenstein TL , Benfield T , Jorgensen NR , Kronborg G , Gerstoft J , Obel N , Kjaer A , Lebech AM . Plasma plasminogen activator inhibitor‐1 predicts myocardial infarction in HIV‐1‐infected individuals. AIDS. 2014;28:1171–1179. 24566095 10.1097/QAD.0000000000000247

[21] Yano Y , Hoshide S , Shimada K , Kario K . The impact of cigarette smoking on 24‐hour blood pressure, inflammatory and hemostatic activity, and cardiovascular risk in Japanese hypertensive patients. J Clin Hypertens. 2013;15:234–240. 10.1111/jch.12047 PMC8033804 23551722

[22] De Luca A , de Gaetano Donati K , Colafigli M , Cozzi‐Lepri A , De Curtis A , Gori A , Sighinolfi L , Giacometti A , Capobianchi MR , D'Avino A , Iacoviello L , Cauda R , D'Arminio Monforte A . The association of high‐sensitivity C‐reactive protein and other biomarkers with cardiovascular disease in patients treated for HIV: a nested case‐control study. BMC Infect Dis. 2013;13:414. 24004495 10.1186/1471-2334-13-414 PMC3846422

[23] Aleksic N , Wang YW , Ahn C , Juneja HS , Folsom AR , Wu KK . Assessment of coronary heart disease risk by combined analysis of coagulation factors. Atherosclerosis. 2008;198:294–300. 18342864 10.1016/j.atherosclerosis.2007.12.062 PMC2579959

[24] Brazionis L , Rowley K , Jenkins A , Itsiopoulos C , O'Dea K . Plasminogen activator inhibitor‐1 activity in type 2 diabetes: a different relationship with coronary heart disease and diabetic retinopathy. Arterioscler Thromb Vasc Biol. 2008;28:786–791. 18239151 10.1161/ATVBAHA.107.160168

[25] Burgess S , Butterworth A , Thompson SG . Mendelian randomization analysis with multiple genetic variants using summarized data. Genet Epidemiol. 2013;37:658–665. 24114802 10.1002/gepi.21758 PMC4377079

[26] Fall T , Hagg S , Magi R , Ploner A , Fischer K , Horikoshi M , Sarin AP , Thorleifsson G , Ladenvall C , Kals M , Kuningas M , Draisma HH , Ried JS , van Zuydam NR , Huikari V , Mangino M , Sonestedt E , Benyamin B , Nelson CP , Rivera NV , Kristiansson K , Shen HY , Havulinna AS , Dehghan A , Donnelly LA , Kaakinen M , Nuotio ML , Robertson N , de Bruijn RF , Ikram MA , Amin N , Balmforth AJ , Braund PS , Doney AS , Doring A , Elliott P , Esko T , Franco OH , Gretarsdottir S , Hartikainen AL , Heikkila K , Herzig KH , Holm H , Hottenga JJ , Hypponen E , Illig T , Isaacs A , Isomaa B , Karssen LC , Kettunen J , Koenig W , Kuulasmaa K , Laatikainen T , Laitinen J , Lindgren C , Lyssenko V , Laara E , Rayner NW , Mannisto S , Pouta A , Rathmann W , Rivadeneira F , Ruokonen A , Savolainen MJ , Sijbrands EJ , Small KS , Smit JH , Steinthorsdottir V , Syvanen AC , Taanila A , Tobin MD , Uitterlinden AG , Willems SM , Willemsen G , Witteman J , Perola M , Evans A , Ferrieres J , Virtamo J , Kee F , Tregouet DA , Arveiler D , Amouyel P , Ferrario MM , Brambilla P , Hall AS , Heath AC , Madden PA , Martin NG , Montgomery GW , Whitfield JB , Jula A , Knekt P , Oostra B , van Duijn CM , Penninx BW , Davey Smith G , Kaprio J , Samani NJ , Gieger C , Peters A , Wichmann HE , Boomsma DI , de Geus EJ , Tuomi T , Power C , Hammond CJ , Spector TD , Lind L , Orho‐Melander M , Palmer CN , Morris AD , Groop L , Jarvelin MR , Salomaa V , Vartiainen E , Hofman A , Ripatti S , Metspalu A , Thorsteinsdottir U , Stefansson K , Pedersen NL , McCarthy MI , Ingelsson E , Prokopenko I ; European Network for G, Genomic Epidemiology c . The role of adiposity in cardiometabolic traits: a Mendelian randomization analysis. PLoS Med. 2013;10:e1001474. 23824655 10.1371/journal.pmed.1001474 PMC3692470

[27] Nikolopoulos GK , Bagos PG , Tsangaris I , Tsiara CG , Kopterides P , Vaiopoulos A , Kapsimali V , Bonovas S , Tsantes AE . The association between plasminogen activator inhibitor type 1 (PAI‐1) levels, PAI‐1 4G/5G polymorphism, and myocardial infarction: a Mendelian randomization meta‐analysis. Clin Chem Lab Med. 2014;52:937–950. 24695040 10.1515/cclm-2013-1124

[28] Huang J , Sabater‐Lleal M , Asselbergs FW , Tregouet D , Shin SY , Ding J , Baumert J , Oudot‐Mellakh T , Folkersen L , Johnson AD , Smith NL , Williams SM , Ikram MA , Kleber ME , Becker DM , Truong V , Mychaleckyj JC , Tang W , Yang Q , Sennblad B , Moore JH , Williams FM , Dehghan A , Silbernagel G , Schrijvers EM , Smith S , Karakas M , Tofler GH , Silveira A , Navis GJ , Lohman K , Chen MH , Peters A , Goel A , Hopewell JC , Chambers JC , Saleheen D , Lundmark P , Psaty BM , Strawbridge RJ , Boehm BO , Carter AM , Meisinger C , Peden JF , Bis JC , McKnight B , Ohrvik J , Taylor K , Franzosi MG , Seedorf U , Collins R , Franco‐Cereceda A , Syvanen AC , Goodall AH , Yanek LR , Cushman M , Muller‐Nurasyid M , Folsom AR , Basu S , Matijevic N , van Gilst WH , Kooner JS , Hofman A , Danesh J , Clarke R , Meigs JB ; Consortium D , Kathiresan S , Reilly MP ; Consortium CA , Klopp N , Harris TB , Winkelmann BR , Grant PJ , Hillege HL , Watkins H ; Consortium CD , Spector TD , Becker LC , Tracy RP , Marz W , Uitterlinden AG , Eriksson P , Cambien F ; Consortium C , Morange PE , Koenig W , Soranzo N , van der Harst P , Liu Y , O'Donnell CJ , Hamsten A . Genome‐wide association study for circulating levels of PAI‐1 provides novel insights into its regulation. Blood. 2012;120:4873–4881. 22990020 10.1182/blood-2012-06-436188 PMC3520624

[29] Ding EL , Song Y , Manson JE , Hunter DJ , Lee CC , Rifai N , Buring JE , Gaziano JM , Liu S . Sex hormone‐binding globulin and risk of type 2 diabetes in women and men. N Engl J Med. 2009;361:1152–1163. 19657112 10.1056/NEJMoa0804381 PMC2774225

[30] Hagg S , Fall T , Ploner A , Magi R , Fischer K , Draisma HH , Kals M , de Vries PS , Dehghan A , Willems SM , Sarin AP , Kristiansson K , Nuotio ML , Havulinna AS , de Bruijn RF , Ikram MA , Kuningas M , Stricker BH , Franco OH , Benyamin B , Gieger C , Hall AS , Huikari V , Jula A , Jarvelin MR , Kaakinen M , Kaprio J , Kobl M , Mangino M , Nelson CP , Palotie A , Samani NJ , Spector TD , Strachan DP , Tobin MD , Whitfield JB , Uitterlinden AG , Salomaa V , Syvanen AC , Kuulasmaa K , Magnusson PK , Esko T , Hofman A , de Geus EJ , Lind L , Giedraitis V , Perola M , Evans A , Ferrieres J , Virtamo J , Kee F , Tregouet DA , Arveiler D , Amouyel P , Gianfagna F , Brambilla P , Ripatti S , van Duijn CM , Metspalu A , Prokopenko I , McCarthy MI , Pedersen NL , Ingelsson E ; European Network for G, Genomic Epidemiology C . Adiposity as a cause of cardiovascular disease: a Mendelian randomization study. Int J Epidemiol. 2015;44:578–586. 26016847 10.1093/ije/dyv094 PMC4553708

[31] Luc G , Empana JP , Morange P , Juhan‐Vague I , Arveiler D , Ferrieres J , Amouyel P , Evans A , Kee F , Bingham A , Machez E , Ducimetiere P . Adipocytokines and the risk of coronary heart disease in healthy middle aged men: the PRIME Study. Int J Obes. 2010;34:118–126. 10.1038/ijo.2009.204 19823188

[32] Burgess S , Timpson NJ , Ebrahim S , Davey Smith G . Mendelian randomization: where are we now and where are we going? Int J Epidemiol. 2015;44:379–388. 26085674 10.1093/ije/dyv108

[33] Bis JC , Kavousi M , Franceschini N , Isaacs A , Abecasis GR , Schminke U , Post WS , Smith AV , Cupples LA , Markus HS , Schmidt R , Huffman JE , Lehtimaki T , Baumert J , Munzel T , Heckbert SR , Dehghan A , North K , Oostra B , Bevan S , Stoegerer EM , Hayward C , Raitakari O , Meisinger C , Schillert A , Sanna S , Volzke H , Cheng YC , Thorsson B , Fox CS , Rice K , Rivadeneira F , Nambi V , Halperin E , Petrovic KE , Peltonen L , Wichmann HE , Schnabel RB , Dorr M , Parsa A , Aspelund T , Demissie S , Kathiresan S , Reilly MP , Taylor K , Uitterlinden A , Couper DJ , Sitzer M , Kahonen M , Illig T , Wild PS , Orru M , Ludemann J , Shuldiner AR , Eiriksdottir G , White CC , Rotter JI , Hofman A , Seissler J , Zeller T , Usala G , Ernst F , Launer LJ , D'Agostino RB Sr , O'Leary DH , Ballantyne C , Thiery J , Ziegler A , Lakatta EG , Chilukoti RK , Harris TB , Wolf PA , Psaty BM , Polak JF , Li X , Rathmann W , Uda M , Boerwinkle E , Klopp N , Schmidt H , Wilson JF , Viikari J , Koenig W , Blankenberg S , Newman AB , Witteman J , Heiss G , Duijn C , Scuteri A , Homuth G , Mitchell BD , Gudnason V , O'Donnell CJ ; Consortium CA . Meta‐analysis of genome‐wide association studies from the CHARGE consortium identifies common variants associated with carotid intima media thickness and plaque. Nat Genet. 2011;43:940–947. 21909108 10.1038/ng.920 PMC3257519

[34] Dastani Z , Hivert MF , Timpson N , Perry JR , Yuan X , Scott RA , Henneman P , Heid IM , Kizer JR , Lyytikainen LP , Fuchsberger C , Tanaka T , Morris AP , Small K , Isaacs A , Beekman M , Coassin S , Lohman K , Qi L , Kanoni S , Pankow JS , Uh HW , Wu Y , Bidulescu A , Rasmussen‐Torvik LJ , Greenwood CM , Ladouceur M , Grimsby J , Manning AK , Liu CT , Kooner J , Mooser VE , Vollenweider P , Kapur KA , Chambers J , Wareham NJ , Langenberg C , Frants R , Willems‐Vandijk K , Oostra BA , Willems SM , Lamina C , Winkler TW , Psaty BM , Tracy RP , Brody J , Chen I , Viikari J , Kahonen M , Pramstaller PP , Evans DM , St Pourcain B , Sattar N , Wood AR , Bandinelli S , Carlson OD , Egan JM , Bohringer S , van Heemst D , Kedenko L , Kristiansson K , Nuotio ML , Loo BM , Harris T , Garcia M , Kanaya A , Haun M , Klopp N , Wichmann HE , Deloukas P , Katsareli E , Couper DJ , Duncan BB , Kloppenburg M , Adair LS , Borja JB ; Consortium D, Consortium M, Investigators G, Mu TC , Wilson JG , Musani S , Guo X , Johnson T , Semple R , Teslovich TM , Allison MA , Redline S , Buxbaum SG , Mohlke KL , Meulenbelt I , Ballantyne CM , Dedoussis GV , Hu FB , Liu Y , Paulweber B , Spector TD , Slagboom PE , Ferrucci L , Jula A , Perola M , Raitakari O , Florez JC , Salomaa V , Eriksson JG , Frayling TM , Hicks AA , Lehtimaki T , Smith GD , Siscovick DS , Kronenberg F , van Duijn C , Loos RJ , Waterworth DM , Meigs JB , Dupuis J , Richards JB , Voight BF , Scott LJ , Steinthorsdottir V , Dina C , Welch RP , Zeggini E , Huth C , Aulchenko YS , Thorleifsson G , McCulloch LJ , Ferreira T , Grallert H , Amin N , Wu G , Willer CJ , Raychaudhuri S , McCarroll SA , Hofmann OM , Segre AV , van Hoek M , Navarro P , Ardlie K , Balkau B , Benediktsson R , Bennett AJ , Blagieva R , Boerwinkle E , Bonnycastle LL , Bostrom KB , Bravenboer B , Bumpstead S , Burtt NP , Charpentier G , Chines PS , Cornelis M , Crawford G , Doney AS , Elliott KS , Elliott AL , Erdos MR , Fox CS , Franklin CS , Ganser M , Gieger C , Grarup N , Green T , Griffin S , Groves CJ , Guiducci C , Hadjadj S , Hassanali N , Herder C , Isomaa B , Jackson AU , Johnson PR , Jorgensen T , Kao WH , Kong A , Kraft P , Kuusisto J , Lauritzen T , Li M , Lieverse A , Lindgren CM , Lyssenko V , Marre M , Meitinger T , Midthjell K , Morken MA , Narisu N , Nilsson P , Owen KR , Payne F , Petersen AK , Platou C , Proenca C , Prokopenko I , Rathmann W , Rayner NW , Robertson NR , Rocheleau G , Roden M , Sampson MJ , Saxena R , Shields BM , Shrader P , Sigurdsson G , Sparso T , Strassburger K , Stringham HM , Sun Q , Swift AJ , Thorand B , Tichet J , Tuomi T , van Dam RM , van Haeften TW , van Herpt T , van Vliet‐Ostaptchouk JV , Walters GB , Weedon MN , Wijmenga C , Witteman J , Bergman RN , Cauchi S , Collins FS , Gloyn AL , Gyllensten U , Hansen T , Hide WA , Hitman GA , Hofman A , Hunter DJ , Hveem K , Laakso M , Morris AD , Palmer CN , Rudan I , Sijbrands E , Stein LD , Tuomilehto J , Uitterlinden A , Walker M , Watanabe RM , Abecasis GR , Boehm BO , Campbell H , Daly MJ , Hattersley AT , Pedersen O , Barroso I , Groop L , Sladek R , Thorsteinsdottir U , Wilson JF , Illig T , Froguel P , van Duijn CM , Stefansson K , Altshuler D , Boehnke M , McCarthy MI , Soranzo N , Wheeler E , Glazer NL , Bouatia‐Naji N , Magi R , Randall J , Elliott P , Rybin D , Dehghan A , Hottenga JJ , Song K , Goel A , Lajunen T , Doney A , Cavalcanti‐Proenca C , Kumari M , Timpson NJ , Zabena C , Ingelsson E , An P , O'Connell J , Luan J , Elliott A , McCarroll SA , Roccasecca RM , Pattou F , Sethupathy P , Ariyurek Y , Barter P , Beilby JP , Ben‐Shlomo Y , Bergmann S , Bochud M , Bonnefond A , Borch‐Johnsen K , Bottcher Y , Brunner E , Bumpstead SJ , Chen YD , Chines P , Clarke R , Coin LJ , Cooper MN , Crisponi L , Day IN , de Geus EJ , Delplanque J , Fedson AC , Fischer‐Rosinsky A , Forouhi NG , Franzosi MG , Galan P , Goodarzi MO , Graessler J , Grundy S , Gwilliam R , Hallmans G , Hammond N , Han X , Hartikainen AL , Hayward C , Heath SC , Hercberg S , Hillman DR , Hingorani AD , Hui J , Hung J , Kaakinen M , Kaprio J , Kesaniemi YA , Kivimaki M , Knight B , Koskinen S , Kovacs P , Kyvik KO , Lathrop GM , Lawlor DA , Le Bacquer O , Lecoeur C , Li Y , Mahley R , Mangino M , Martinez‐Larrad MT , McAteer JB , McPherson R , Meisinger C , Melzer D , Meyre D , Mitchell BD , Mukherjee S , Naitza S , Neville MJ , Orru M , Pakyz R , Paolisso G , Pattaro C , Pearson D , Peden JF , Pedersen NL , Pfeiffer AF , Pichler I , Polasek O , Posthuma D , Potter SC , Pouta A , Province MA , Rayner NW , Rice K , Ripatti S , Rivadeneira F , Rolandsson O , Sandbaek A , Sandhu M , Sanna S , Sayer AA , Scheet P , Seedorf U , Sharp SJ , Shields B , Sigurethsson G , Sijbrands EJ , Silveira A , Simpson L , Singleton A , Smith NL , Sovio U , Swift A , Syddall H , Syvanen AC , Tonjes A , Uitterlinden AG , van Dijk KW , Varma D , Visvikis‐Siest S , Vitart V , Vogelzangs N , Waeber G , Wagner PJ , Walley A , Ward KL , Watkins H , Wild SH , Willemsen G , Witteman JC , Yarnell JW , Zelenika D , Zethelius B , Zhai G , Zhao JH , Zillikens MC ; Consortium D, Consortium G, Global BPC , Borecki IB , Meneton P , Magnusson PK , Nathan DM , Williams GH , Silander K , Bornstein SR , Schwarz P , Spranger J , Karpe F , Shuldiner AR , Cooper C , Serrano‐Rios M , Lind L , Palmer LJ , Hu FBs , Franks PW , Ebrahim S , Marmot M , Kao WH , Pramstaller PP , Wright AF , Stumvoll M , Hamsten A ; Procardis C , Buchanan TA , Valle TT , Rotter JI , Penninx BW , Boomsma DI , Cao A , Scuteri A , Schlessinger D , Uda M , Ruokonen A , Jarvelin MR , Peltonen L , Mooser V , Sladek R ; Investigators M, Consortium G , Musunuru K , Smith AV , Edmondson AC , Stylianou IM , Koseki M , Pirruccello JP , Chasman DI , Johansen CT , Fouchier SW , Peloso GM , Barbalic M , Ricketts SL , Bis JC , Feitosa MF , Orho‐Melander M , Melander O , Li X , Li M , Cho YS , Go MJ , Kim YJ , Lee JY , Park T , Kim K , Sim X , Ong RT , Croteau‐Chonka DC , Lange LA , Smith JD , Ziegler A , Zhang W , Zee RY , Whitfield JB , Thompson JR , Surakka I , Spector TD , Smit JH , Sinisalo J , Scott J , Saharinen J , Sabatti C , Rose LM , Roberts R , Rieder M , Parker AN , Pare G , O'Donnell CJ , Nieminen MS , Nickerson DA , Montgomery GW , McArdle W , Masson D , Martin NG , Marroni F , Lucas G , Luben R , Lokki ML , Lettre G , Launer LJ , Lakatta EG , Laaksonen R , Kyvik KO , Konig IR , Khaw KT , Kaplan LM , Johansson A , Janssens AC , Igl W , Hovingh GK , Hengstenberg C , Havulinna AS , Hastie ND , Harris TB , Haritunians T , Hall AS , Groop LC , Gonzalez E , Freimer NB , Erdmann J , Ejebe KG , Doring A , Dominiczak AF , Demissie S , Deloukas P , de Faire U , Crawford G , Chen YD , Caulfield MJ , Boekholdt SM , Assimes TL , Quertermous T , Seielstad M , Wong TY , Tai ES , Feranil AB , Kuzawa CW , Taylor HA Jr , Gabriel SB , Holm H , Gudnason V , Krauss RM , Ordovas JM , Munroe PB , Kooner JS , Tall AR , Hegele RA , Kastelein JJ , Schadt EE , Strachan DP , Reilly MP , Samani NJ , Schunkert H , Cupples LA , Sandhu MS , Ridker PM , Rader DJ , Kathiresan S . Novel loci for adiponectin levels and their influence on type 2 diabetes and metabolic traits: a multi‐ethnic meta‐analysis of 45,891 individuals. PLoS Genet. 2012;8:e1002607. 22479202 10.1371/journal.pgen.1002607 PMC3315470

[35] Ehret GB , Munroe PB , Rice KM , Bochud M , Johnson AD , Chasman DI , Smith AV , Tobin MD , Verwoert GC , Hwang SJ , Pihur V , Vollenweider P , O'Reilly PF , Amin N , Bragg‐Gresham JL , Teumer A , Glazer NL , Launer L , Zhao JH , Aulchenko Y , Heath S , Sober S , Parsa A , Luan J , Arora P , Dehghan A , Zhang F , Lucas G , Hicks AA , Jackson AU , Peden JF , Tanaka T , Wild SH , Rudan I , Igl W , Milaneschi Y , Parker AN , Fava C , Chambers JC , Fox ER , Kumari M , Go MJ , van der Harst P , Kao WH , Sjogren M , Vinay DG , Alexander M , Tabara Y , Shaw‐Hawkins S , Whincup PH , Liu Y , Shi G , Kuusisto J , Tayo B , Seielstad M , Sim X , Nguyen KD , Lehtimaki T , Matullo G , Wu Y , Gaunt TR , Onland‐Moret NC , Cooper MN , Platou CG , Org E , Hardy R , Dahgam S , Palmen J , Vitart V , Braund PS , Kuznetsova T , Uiterwaal CS , Adeyemo A , Palmas W , Campbell H , Ludwig B , Tomaszewski M , Tzoulaki I , Palmer ND ; Consortium CA, Consortium CK, KidneyGen C, EchoGen c, consortium C‐H , Aspelund T , Garcia M , Chang YP , O'Connell JR , Steinle NI , Grobbee DE , Arking DE , Kardia SL , Morrison AC , Hernandez D , Najjar S , McArdle WL , Hadley D , Brown MJ , Connell JM , Hingorani AD , Day IN , Lawlor DA , Beilby JP , Lawrence RW , Clarke R , Hopewell JC , Ongen H , Dreisbach AW , Li Y , Young JH , Bis JC , Kahonen M , Viikari J , Adair LS , Lee NR , Chen MH , Olden M , Pattaro C , Bolton JA , Kottgen A , Bergmann S , Mooser V , Chaturvedi N , Frayling TM , Islam M , Jafar TH , Erdmann J , Kulkarni SR , Bornstein SR , Grassler J , Groop L , Voight BF , Kettunen J , Howard P , Taylor A , Guarrera S , Ricceri F , Emilsson V , Plump A , Barroso I , Khaw KT , Weder AB , Hunt SC , Sun YV , Bergman RN , Collins FS , Bonnycastle LL , Scott LJ , Stringham HM , Peltonen L , Perola M , Vartiainen E , Brand SM , Staessen JA , Wang TJ , Burton PR , Soler Artigas M , Dong Y , Snieder H , Wang X , Zhu H , Lohman KK , Rudock ME , Heckbert SR , Smith NL , Wiggins KL , Doumatey A , Shriner D , Veldre G , Viigimaa M , Kinra S , Prabhakaran D , Tripathy V , Langefeld CD , Rosengren A , Thelle DS , Corsi AM , Singleton A , Forrester T , Hilton G , McKenzie CA , Salako T , Iwai N , Kita Y , Ogihara T , Ohkubo T , Okamura T , Ueshima H , Umemura S , Eyheramendy S , Meitinger T , Wichmann HE , Cho YS , Kim HL , Lee JY , Scott J , Sehmi JS , Zhang W , Hedblad B , Nilsson P , Smith GD , Wong A , Narisu N , Stancakova A , Raffel LJ , Yao J , Kathiresan S , O'Donnell CJ , Schwartz SM , Ikram MA , Longstreth WT Jr , Mosley TH , Seshadri S , Shrine NR , Wain LV , Morken MA , Swift AJ , Laitinen J , Prokopenko I , Zitting P , Cooper JA , Humphries SE , Danesh J , Rasheed A , Goel A , Hamsten A , Watkins H , Bakker SJ , van Gilst WH , Janipalli CS , Mani KR , Yajnik CS , Hofman A , Mattace‐Raso FU , Oostra BA , Demirkan A , Isaacs A , Rivadeneira F , Lakatta EG , Orru M , Scuteri A , Ala‐Korpela M , Kangas AJ , Lyytikainen LP , Soininen P , Tukiainen T , Wurtz P , Ong RT , Dorr M , Kroemer HK , Volker U , Volzke H , Galan P , Hercberg S , Lathrop M , Zelenika D , Deloukas P , Mangino M , Spector TD , Zhai G , Meschia JF , Nalls MA , Sharma P , Terzic J , Kumar MV , Denniff M , Zukowska‐Szczechowska E , Wagenknecht LE , Fowkes FG , Charchar FJ , Schwarz PE , Hayward C , Guo X , Rotimi C , Bots ML , Brand E , Samani NJ , Polasek O , Talmud PJ , Nyberg F , Kuh D , Laan M , Hveem K , Palmer LJ , van der Schouw YT , Casas JP , Mohlke KL , Vineis P , Raitakari O , Ganesh SK , Wong TY , Tai ES , Cooper RS , Laakso M , Rao DC , Harris TB , Morris RW , Dominiczak AF , Kivimaki M , Marmot MG , Miki T , Saleheen D , Chandak GR , Coresh J , Navis G , Salomaa V , Han BG , Zhu X , Kooner JS , Melander O , Ridker PM , Bandinelli S , Gyllensten UB , Wright AF , Wilson JF , Ferrucci L , Farrall M , Tuomilehto J , Pramstaller PP , Elosua R , Soranzo N , Sijbrands EJ , Altshuler D , Loos RJ , Shuldiner AR , Gieger C , Meneton P , Uitterlinden AG , Wareham NJ , Gudnason V , Rotter JI , Rettig R , Uda M , Strachan DP , Witteman JC , Hartikainen AL , Beckmann JS , Boerwinkle E , Vasan RS , Boehnke M , Larson MG , Jarvelin MR , Psaty BM , Abecasis GR , Chakravarti A , Elliott P , van Duijn CM , Newton‐Cheh C , Levy D , Caulfield MJ , Johnson T . Genetic variants in novel pathways influence blood pressure and cardiovascular disease risk. Nature. 2011;478:103–109. 21909115 10.1038/nature10405 PMC3340926

[36] Locke AE , Kahali B , Berndt SI , Justice AE , Pers TH , Day FR , Powell C , Vedantam S , Buchkovich ML , Yang J , Croteau‐Chonka DC , Esko T , Fall T , Ferreira T , Gustafsson S , Kutalik Z , Luan J , Magi R , Randall JC , Winkler TW , Wood AR , Workalemahu T , Faul JD , Smith JA , Hua Zhao J , Zhao W , Chen J , Fehrmann R , Hedman AK , Karjalainen J , Schmidt EM , Absher D , Amin N , Anderson D , Beekman M , Bolton JL , Bragg‐Gresham JL , Buyske S , Demirkan A , Deng G , Ehret GB , Feenstra B , Feitosa MF , Fischer K , Goel A , Gong J , Jackson AU , Kanoni S , Kleber ME , Kristiansson K , Lim U , Lotay V , Mangino M , Mateo Leach I , Medina‐Gomez C , Medland SE , Nalls MA , Palmer CD , Pasko D , Pechlivanis S , Peters MJ , Prokopenko I , Shungin D , Stancakova A , Strawbridge RJ , Ju Sung Y , Tanaka T , Teumer A , Trompet S , van der Laan SW , van Setten J , Van Vliet‐Ostaptchouk JV , Wang Z , Yengo L , Zhang W , Isaacs A , Albrecht E , Arnlov J , Arscott GM , Attwood AP , Bandinelli S , Barrett A , Bas IN , Bellis C , Bennett AJ , Berne C , Blagieva R , Bluher M , Bohringer S , Bonnycastle LL , Bottcher Y , Boyd HA , Bruinenberg M , Caspersen IH , Ida Chen YD , Clarke R , Daw EW , de Craen AJ , Delgado G , Dimitriou M , Doney AS , Eklund N , Estrada K , Eury E , Folkersen L , Fraser RM , Garcia ME , Geller F , Giedraitis V , Gigante B , Go AS , Golay A , Goodall AH , Gordon SD , Gorski M , Grabe HJ , Grallert H , Grammer TB , Grassler J , Gronberg H , Groves CJ , Gusto G , Haessler J , Hall P , Haller T , Hallmans G , Hartman CA , Hassinen M , Hayward C , Heard‐Costa NL , Helmer Q , Hengstenberg C , Holmen O , Hottenga JJ , James AL , Jeff JM , Johansson A , Jolley J , Juliusdottir T , Kinnunen L , Koenig W , Koskenvuo M , Kratzer W , Laitinen J , Lamina C , Leander K , Lee NR , Lichtner P , Lind L , Lindstrom J , Sin Lo K , Lobbens S , Lorbeer R , Lu Y , Mach F , Magnusson PK , Mahajan A , McArdle WL , McLachlan S , Menni C , Merger S , Mihailov E , Milani L , Moayyeri A , Monda KL , Morken MA , Mulas A , Muller G , Muller‐Nurasyid M , Musk AW , Nagaraja R , Nothen MM , Nolte IM , Pilz S , Rayner NW , Renstrom F , Rettig R , Ried JS , Ripke S , Robertson NR , Rose LM , Sanna S , Scharnagl H , Scholtens S , Schumacher FR , Scott WR , Seufferlein T , Shi J , Vernon Smith A , Smolonska J , Stanton AV , Steinthorsdottir V , Stirrups K , Stringham HM , Sundstrom J , Swertz MA , Swift AJ , Syvanen AC , Tan ST , Tayo BO , Thorand B , Thorleifsson G , Tyrer JP , Uh HW , Vandenput L , Verhulst FC , Vermeulen SH , Verweij N , Vonk JM , Waite LL , Warren HR , Waterworth D , Weedon MN , Wilkens LR , Willenborg C , Wilsgaard T , Wojczynski MK , Wong A , Wright AF , Zhang Q ; LifeLines Cohort S , Brennan EP , Choi M , Dastani Z , Drong AW , Eriksson P , Franco‐Cereceda A , Gadin JR , Gharavi AG , Goddard ME , Handsaker RE , Huang J , Karpe F , Kathiresan S , Keildson S , Kiryluk K , Kubo M , Lee JY , Liang L , Lifton RP , Ma B , McCarroll SA , McKnight AJ , Min JL , Moffatt MF , Montgomery GW , Murabito JM , Nicholson G , Nyholt DR , Okada Y , Perry JR , Dorajoo R , Reinmaa E , Salem RM , Sandholm N , Scott RA , Stolk L , Takahashi A , Tanaka T , Van't Hooft FM , Vinkhuyzen AA , Westra HJ , Zheng W , Zondervan KT ; Consortium AD, Group A‐BW, Consortium CAD, Consortium CK, Glgc, Icbp, Investigators M, Mu TC, Consortium MI, Consortium P, ReproGen C, Consortium G, International Endogene C , Heath AC , Arveiler D , Bakker SJ , Beilby J , Bergman RN , Blangero J , Bovet P , Campbell H , Caulfield MJ , Cesana G , Chakravarti A , Chasman DI , Chines PS , Collins FS , Crawford DC , Cupples LA , Cusi D , Danesh J , de Faire U , den Ruijter HM , Dominiczak AF , Erbel R , Erdmann J , Eriksson JG , Farrall M , Felix SB , Ferrannini E , Ferrieres J , Ford I , Forouhi NG , Forrester T , Franco OH , Gansevoort RT , Gejman PV , Gieger C , Gottesman O , Gudnason V , Gyllensten U , Hall AS , Harris TB , Hattersley AT , Hicks AA , Hindorff LA , Hingorani AD , Hofman A , Homuth G , Hovingh GK , Humphries SE , Hunt SC , Hypponen E , Illig T , Jacobs KB , Jarvelin MR , Jockel KH , Johansen B , Jousilahti P , Jukema JW , Jula AM , Kaprio J , Kastelein JJ , Keinanen‐Kiukaanniemi SM , Kiemeney LA , Knekt P , Kooner JS , Kooperberg C , Kovacs P , Kraja AT , Kumari M , Kuusisto J , Lakka TA , Langenberg C , Le Marchand L , Lehtimaki T , Lyssenko V , Mannisto S , Marette A , Matise TC , McKenzie CA , McKnight B , Moll FL , Morris AD , Morris AP , Murray JC , Nelis M , Ohlsson C , Oldehinkel AJ , Ong KK , Madden PA , Pasterkamp G , Peden JF , Peters A , Postma DS , Pramstaller PP , Price JF , Qi L , Raitakari OT , Rankinen T , Rao DC , Rice TK , Ridker PM , Rioux JD , Ritchie MD , Rudan I , Salomaa V , Samani NJ , Saramies J , Sarzynski MA , Schunkert H , Schwarz PE , Sever P , Shuldiner AR , Sinisalo J , Stolk RP , Strauch K , Tonjes A , Tregouet DA , Tremblay A , Tremoli E , Virtamo J , Vohl MC , Volker U , Waeber G , Willemsen G , Witteman JC , Zillikens MC , Adair LS , Amouyel P , Asselbergs FW , Assimes TL , Bochud M , Boehm BO , Boerwinkle E , Bornstein SR , Bottinger EP , Bouchard C , Cauchi S , Chambers JC , Chanock SJ , Cooper RS , de Bakker PI , Dedoussis G , Ferrucci L , Franks PW , Froguel P , Groop LC , Haiman CA , Hamsten A , Hui J , Hunter DJ , Hveem K , Kaplan RC , Kivimaki M , Kuh D , Laakso M , Liu Y , Martin NG , Marz W , Melbye M , Metspalu A , Moebus S , Munroe PB , Njolstad I , Oostra BA , Palmer CN , Pedersen NL , Perola M , Perusse L , Peters U , Power C , Quertermous T , Rauramaa R , Rivadeneira F , Saaristo TE , Saleheen D , Sattar N , Schadt EE , Schlessinger D , Slagboom PE , Snieder H , Spector TD , Thorsteinsdottir U , Stumvoll M , Tuomilehto J , Uitterlinden AG , Uusitupa M , van der Harst P , Walker M , Wallaschofski H , Wareham NJ , Watkins H , Weir DR , Wichmann HE , Wilson JF , Zanen P , Borecki IB , Deloukas P , Fox CS , Heid IM , O'Connell JR , Strachan DP , Stefansson K , van Duijn CM , Abecasis GR , Franke L , Frayling TM , McCarthy MI , Visscher PM , Scherag A , Willer CJ , Boehnke M , Mohlke KL , Lindgren CM , Beckmann JS , Barroso I , North KE , Ingelsson E , Hirschhorn JN , Loos RJ , Speliotes EK . Genetic studies of body mass index yield new insights for obesity biology. Nature. 2015;518:197–206. 25673413 10.1038/nature14177 PMC4382211

[37] Manning AK , Hivert MF , Scott RA , Grimsby JL , Bouatia‐Naji N , Chen H , Rybin D , Liu CT , Bielak LF , Prokopenko I , Amin N , Barnes D , Cadby G , Hottenga JJ , Ingelsson E , Jackson AU , Johnson T , Kanoni S , Ladenvall C , Lagou V , Lahti J , Lecoeur C , Liu Y , Martinez‐Larrad MT , Montasser ME , Navarro P , Perry JR , Rasmussen‐Torvik LJ , Salo P , Sattar N , Shungin D , Strawbridge RJ , Tanaka T , van Duijn CM , An P , de Andrade M , Andrews JS , Aspelund T , Atalay M , Aulchenko Y , Balkau B , Bandinelli S , Beckmann JS , Beilby JP , Bellis C , Bergman RN , Blangero J , Boban M , Boehnke M , Boerwinkle E , Bonnycastle LL , Boomsma DI , Borecki IB , Bottcher Y , Bouchard C , Brunner E , Budimir D , Campbell H , Carlson O , Chines PS , Clarke R , Collins FS , Corbaton‐Anchuelo A , Couper D , de Faire U , Dedoussis GV , Deloukas P , Dimitriou M , Egan JM , Eiriksdottir G , Erdos MR , Eriksson JG , Eury E , Ferrucci L , Ford I , Forouhi NG , Fox CS , Franzosi MG , Franks PW , Frayling TM , Froguel P , Galan P , de Geus E , Gigante B , Glazer NL , Goel A , Groop L , Gudnason V , Hallmans G , Hamsten A , Hansson O , Harris TB , Hayward C , Heath S , Hercberg S , Hicks AA , Hingorani A , Hofman A , Hui J , Hung J , Jarvelin MR , Jhun MA , Johnson PC , Jukema JW , Jula A , Kao WH , Kaprio J , Kardia SL , Keinanen‐Kiukaanniemi S , Kivimaki M , Kolcic I , Kovacs P , Kumari M , Kuusisto J , Kyvik KO , Laakso M , Lakka T , Lannfelt L , Lathrop GM , Launer LJ , Leander K , Li G , Lind L , Lindstrom J , Lobbens S , Loos RJ , Luan J , Lyssenko V , Magi R , Magnusson PK , Marmot M , Meneton P , Mohlke KL , Mooser V , Morken MA , Miljkovic I , Narisu N , O'Connell J , Ong KK , Oostra BA , Palmer LJ , Palotie A , Pankow JS , Peden JF , Pedersen NL , Pehlic M , Peltonen L , Penninx B , Pericic M , Perola M , Perusse L , Peyser PA , Polasek O , Pramstaller PP , Province MA , Raikkonen K , Rauramaa R , Rehnberg E , Rice K , Rotter JI , Rudan I , Ruokonen A , Saaristo T , Sabater‐Lleal M , Salomaa V , Savage DB , Saxena R , Schwarz P , Seedorf U , Sennblad B , Serrano‐Rios M , Shuldiner AR , Sijbrands EJ , Siscovick DS , Smit JH , Small KS , Smith NL , Smith AV , Stancakova A , Stirrups K , Stumvoll M , Sun YV , Swift AJ , Tonjes A , Tuomilehto J , Trompet S , Uitterlinden AG , Uusitupa M , Vikstrom M , Vitart V , Vohl MC , Voight BF , Vollenweider P , Waeber G , Waterworth DM , Watkins H , Wheeler E , Widen E , Wild SH , Willems SM , Willemsen G , Wilson JF , Witteman JC , Wright AF , Yaghootkar H , Zelenika D , Zemunik T , Zgaga L ; Replication DIG, Meta‐analysis C, Multiple Tissue Human Expression Resource C , Wareham NJ , McCarthy MI , Barroso I , Watanabe RM , Florez JC , Dupuis J , Meigs JB , Langenberg C . A genome‐wide approach accounting for body mass index identifies genetic variants influencing fasting glycemic traits and insulin resistance. Nat Genet. 2012;44:659–669. 22581228 10.1038/ng.2274 PMC3613127

[38] Morris AP , Voight BF , Teslovich TM , Ferreira T , Segre AV , Steinthorsdottir V , Strawbridge RJ , Khan H , Grallert H , Mahajan A , Prokopenko I , Kang HM , Dina C , Esko T , Fraser RM , Kanoni S , Kumar A , Lagou V , Langenberg C , Luan J , Lindgren CM , Muller‐Nurasyid M , Pechlivanis S , Rayner NW , Scott LJ , Wiltshire S , Yengo L , Kinnunen L , Rossin EJ , Raychaudhuri S , Johnson AD , Dimas AS , Loos RJ , Vedantam S , Chen H , Florez JC , Fox C , Liu CT , Rybin D , Couper DJ , Kao WH , Li M , Cornelis MC , Kraft P , Sun Q , van Dam RM , Stringham HM , Chines PS , Fischer K , Fontanillas P , Holmen OL , Hunt SE , Jackson AU , Kong A , Lawrence R , Meyer J , Perry JR , Platou CG , Potter S , Rehnberg E , Robertson N , Sivapalaratnam S , Stancakova A , Stirrups K , Thorleifsson G , Tikkanen E , Wood AR , Almgren P , Atalay M , Benediktsson R , Bonnycastle LL , Burtt N , Carey J , Charpentier G , Crenshaw AT , Doney AS , Dorkhan M , Edkins S , Emilsson V , Eury E , Forsen T , Gertow K , Gigante B , Grant GB , Groves CJ , Guiducci C , Herder C , Hreidarsson AB , Hui J , James A , Jonsson A , Rathmann W , Klopp N , Kravic J , Krjutskov K , Langford C , Leander K , Lindholm E , Lobbens S , Mannisto S , Mirza G , Muhleisen TW , Musk B , Parkin M , Rallidis L , Saramies J , Sennblad B , Shah S , Sigurethsson G , Silveira A , Steinbach G , Thorand B , Trakalo J , Veglia F , Wennauer R , Winckler W , Zabaneh D , Campbell H , van Duijn C , Uitterlinden AG , Hofman A , Sijbrands E , Abecasis GR , Owen KR , Zeggini E , Trip MD , Forouhi NG , Syvanen AC , Eriksson JG , Peltonen L , Nothen MM , Balkau B , Palmer CN , Lyssenko V , Tuomi T , Isomaa B , Hunter DJ , Qi L ; Wellcome Trust Case Control C, Meta‐Analyses of G, Insulin‐related traits Consortium I, Genetic Investigation of ATC, Asian Genetic Epidemiology Network‐Type 2 Diabetes C, South Asian Type 2 Diabetes C , Shuldiner AR , Roden M , Barroso I , Wilsgaard T , Beilby J , Hovingh K , Price JF , Wilson JF , Rauramaa R , Lakka TA , Lind L , Dedoussis G , Njolstad I , Pedersen NL , Khaw KT , Wareham NJ , Keinanen‐Kiukaanniemi SM , Saaristo TE , Korpi‐Hyovalti E , Saltevo J , Laakso M , Kuusisto J , Metspalu A , Collins FS , Mohlke KL , Bergman RN , Tuomilehto J , Boehm BO , Gieger C , Hveem K , Cauchi S , Froguel P , Baldassarre D , Tremoli E , Humphries SE , Saleheen D , Danesh J , Ingelsson E , Ripatti S , Salomaa V , Erbel R , Jockel KH , Moebus S , Peters A , Illig T , de Faire U , Hamsten A , Morris AD , Donnelly PJ , Frayling TM , Hattersley AT , Boerwinkle E , Melander O , Kathiresan S , Nilsson PM , Deloukas P , Thorsteinsdottir U , Groop LC , Stefansson K , Hu F , Pankow JS , Dupuis J , Meigs JB , Altshuler D , Boehnke M , McCarthy MI , Replication DIG, Meta‐analysis C . Large‐scale association analysis provides insights into the genetic architecture and pathophysiology of type 2 diabetes. Nat Genet. 2012;44:981–990. 22885922 10.1038/ng.2383 PMC3442244

[39] Nikpay M , Goel A , Won HH , Hall LM , Willenborg C , Kanoni S , Saleheen D , Kyriakou T , Nelson CP , Hopewell JC , Webb TR , Zeng L , Dehghan A , Alver M , Armasu SM , Auro K , Bjonnes A , Chasman DI , Chen S , Ford I , Franceschini N , Gieger C , Grace C , Gustafsson S , Huang J , Hwang SJ , Kim YK , Kleber ME , Lau KW , Lu X , Lu Y , Lyytikainen LP , Mihailov E , Morrison AC , Pervjakova N , Qu L , Rose LM , Salfati E , Saxena R , Scholz M , Smith AV , Tikkanen E , Uitterlinden A , Yang X , Zhang W , Zhao W , de Andrade M , de Vries PS , van Zuydam NR , Anand SS , Bertram L , Beutner F , Dedoussis G , Frossard P , Gauguier D , Goodall AH , Gottesman O , Haber M , Han BG , Huang J , Jalilzadeh S , Kessler T , Konig IR , Lannfelt L , Lieb W , Lind L , Lindgren CM , Lokki ML , Magnusson PK , Mallick NH , Mehra N , Meitinger T , Memon FU , Morris AP , Nieminen MS , Pedersen NL , Peters A , Rallidis LS , Rasheed A , Samuel M , Shah SH , Sinisalo J , Stirrups KE , Trompet S , Wang L , Zaman KS , Ardissino D , Boerwinkle E , Borecki IB , Bottinger EP , Buring JE , Chambers JC , Collins R , Cupples LA , Danesh J , Demuth I , Elosua R , Epstein SE , Esko T , Feitosa MF , Franco OH , Franzosi MG , Granger CB , Gu D , Gudnason V , Hall AS , Hamsten A , Harris TB , Hazen SL , Hengstenberg C , Hofman A , Ingelsson E , Iribarren C , Jukema JW , Karhunen PJ , Kim BJ , Kooner JS , Kullo IJ , Lehtimaki T , Loos RJ , Melander O , Metspalu A , Marz W , Palmer CN , Perola M , Quertermous T , Rader DJ , Ridker PM , Ripatti S , Roberts R , Salomaa V , Sanghera DK , Schwartz SM , Seedorf U , Stewart AF , Stott DJ , Thiery J , Zalloua PA , O'Donnell CJ , Reilly MP , Assimes TL , Thompson JR , Erdmann J , Clarke R , Watkins H , Kathiresan S , McPherson R , Deloukas P , Schunkert H , Samani NJ , Farrall M ; Consortium CAD . A comprehensive 1,000 genomes‐based genome‐wide association meta‐analysis of coronary artery disease. Nat Genet. 2015;47:1121–1130. 26343387 10.1038/ng.3396 PMC4589895

[40] O'Donnell CJ , Kavousi M , Smith AV , Kardia SL , Feitosa MF , Hwang SJ , Sun YV , Province MA , Aspelund T , Dehghan A , Hoffmann U , Bielak LF , Zhang Q , Eiriksdottir G , van Duijn CM , Fox CS , de Andrade M , Kraja AT , Sigurdsson S , Elias‐Smale SE , Murabito JM , Launer LJ , van der Lugt A , Kathiresan S ; Consortium CA , Krestin GP , Herrington DM , Howard TD , Liu Y , Post W , Mitchell BD , O'Connell JR , Shen H , Shuldiner AR , Altshuler D , Elosua R , Salomaa V , Schwartz SM , Siscovick DS , Voight BF , Bis JC , Glazer NL , Psaty BM , Boerwinkle E , Heiss G , Blankenberg S , Zeller T , Wild PS , Schnabel RB , Schillert A , Ziegler A , Munzel TF , White CC , Rotter JI , Nalls M , Oudkerk M , Johnson AD , Newman AB , Uitterlinden AG , Massaro JM , Cunningham J , Harris TB , Hofman A , Peyser PA , Borecki IB , Cupples LA , Gudnason V , Witteman JC . Genome‐wide association study for coronary artery calcification with follow‐up in myocardial infarction. Circulation. 2011;124:2855–2864. 22144573 10.1161/CIRCULATIONAHA.110.974899 PMC3397173

[41] Shungin D , Winkler TW , Croteau‐Chonka DC , Ferreira T , Locke AE , Magi R , Strawbridge RJ , Pers TH , Fischer K , Justice AE , Workalemahu T , Wu JM , Buchkovich ML , Heard‐Costa NL , Roman TS , Drong AW , Song C , Gustafsson S , Day FR , Esko T , Fall T , Kutalik Z , Luan J , Randall JC , Scherag A , Vedantam S , Wood AR , Chen J , Fehrmann R , Karjalainen J , Kahali B , Liu CT , Schmidt EM , Absher D , Amin N , Anderson D , Beekman M , Bragg‐Gresham JL , Buyske S , Demirkan A , Ehret GB , Feitosa MF , Goel A , Jackson AU , Johnson T , Kleber ME , Kristiansson K , Mangino M , Mateo Leach I , Medina‐Gomez C , Palmer CD , Pasko D , Pechlivanis S , Peters MJ , Prokopenko I , Stancakova A , Ju Sung Y , Tanaka T , Teumer A , Van Vliet‐Ostaptchouk JV , Yengo L , Zhang W , Albrecht E , Arnlov J , Arscott GM , Bandinelli S , Barrett A , Bellis C , Bennett AJ , Berne C , Bluher M , Bohringer S , Bonnet F , Bottcher Y , Bruinenberg M , Carba DB , Caspersen IH , Clarke R , Daw EW , Deelen J , Deelman E , Delgado G , Doney AS , Eklund N , Erdos MR , Estrada K , Eury E , Friedrich N , Garcia ME , Giedraitis V , Gigante B , Go AS , Golay A , Grallert H , Grammer TB , Grassler J , Grewal J , Groves CJ , Haller T , Hallmans G , Hartman CA , Hassinen M , Hayward C , Heikkila K , Herzig KH , Helmer Q , Hillege HL , Holmen O , Hunt SC , Isaacs A , Ittermann T , James AL , Johansson I , Juliusdottir T , Kalafati IP , Kinnunen L , Koenig W , Kooner IK , Kratzer W , Lamina C , Leander K , Lee NR , Lichtner P , Lind L , Lindstrom J , Lobbens S , Lorentzon M , Mach F , Magnusson PK , Mahajan A , McArdle WL , Menni C , Merger S , Mihailov E , Milani L , Mills R , Moayyeri A , Monda KL , Mooijaart SP , Muhleisen TW , Mulas A , Muller G , Muller‐Nurasyid M , Nagaraja R , Nalls MA , Narisu N , Glorioso N , Nolte IM , Olden M , Rayner NW , Renstrom F , Ried JS , Robertson NR , Rose LM , Sanna S , Scharnagl H , Scholtens S , Sennblad B , Seufferlein T , Sitlani CM , Vernon Smith A , Stirrups K , Stringham HM , Sundstrom J , Swertz MA , Swift AJ , Syvanen AC , Tayo BO , Thorand B , Thorleifsson G , Tomaschitz A , Troffa C , van Oort FV , Verweij N , Vonk JM , Waite LL , Wennauer R , Wilsgaard T , Wojczynski MK , Wong A , Zhang Q , Hua Zhao J , Brennan EP , Choi M , Eriksson P , Folkersen L , Franco‐Cereceda A , Gharavi AG , Hedman AK , Hivert MF , Huang J , Kanoni S , Karpe F , Keildson S , Kiryluk K , Liang L , Lifton RP , Ma B , McKnight AJ , McPherson R , Metspalu A , Min JL , Moffatt MF , Montgomery GW , Murabito JM , Nicholson G , Nyholt DR , Olsson C , Perry JR , Reinmaa E , Salem RM , Sandholm N , Schadt EE , Scott RA , Stolk L , Vallejo EE , Westra HJ , Zondervan KT ; Consortium AD, Consortium CAD, Consortium CK, Consortium G, Consortium G, Glgc, Icbp, International Endogene C, LifeLines Cohort S, Investigators M, Mu TC, Consortium P, ReproGen C , Amouyel P , Arveiler D , Bakker SJ , Beilby J , Bergman RN , Blangero J , Brown MJ , Burnier M , Campbell H , Chakravarti A , Chines PS , Claudi‐Boehm S , Collins FS , Crawford DC , Danesh J , de Faire U , de Geus EJ , Dorr M , Erbel R , Eriksson JG , Farrall M , Ferrannini E , Ferrieres J , Forouhi NG , Forrester T , Franco OH , Gansevoort RT , Gieger C , Gudnason V , Haiman CA , Harris TB , Hattersley AT , Heliovaara M , Hicks AA , Hingorani AD , Hoffmann W , Hofman A , Homuth G , Humphries SE , Hypponen E , Illig T , Jarvelin MR , Johansen B , Jousilahti P , Jula AM , Kaprio J , Kee F , Keinanen‐Kiukaanniemi SM , Kooner JS , Kooperberg C , Kovacs P , Kraja AT , Kumari M , Kuulasmaa K , Kuusisto J , Lakka TA , Langenberg C , Le Marchand L , Lehtimaki T , Lyssenko V , Mannisto S , Marette A , Matise TC , McKenzie CA , McKnight B , Musk AW , Mohlenkamp S , Morris AD , Nelis M , Ohlsson C , Oldehinkel AJ , Ong KK , Palmer LJ , Penninx BW , Peters A , Pramstaller PP , Raitakari OT , Rankinen T , Rao DC , Rice TK , Ridker PM , Ritchie MD , Rudan I , Salomaa V , Samani NJ , Saramies J , Sarzynski MA , Schwarz PE , Shuldiner AR , Staessen JA , Steinthorsdottir V , Stolk RP , Strauch K , Tonjes A , Tremblay A , Tremoli E , Vohl MC , Volker U , Vollenweider P , Wilson JF , Witteman JC , Adair LS , Bochud M , Boehm BO , Bornstein SR , Bouchard C , Cauchi S , Caulfield MJ , Chambers JC , Chasman DI , Cooper RS , Dedoussis G , Ferrucci L , Froguel P , Grabe HJ , Hamsten A , Hui J , Hveem K , Jockel KH , Kivimaki M , Kuh D , Laakso M , Liu Y , Marz W , Munroe PB , Njolstad I , Oostra BA , Palmer CN , Pedersen NL , Perola M , Perusse L , Peters U , Power C , Quertermous T , Rauramaa R , Rivadeneira F , Saaristo TE , Saleheen D , Sinisalo J , Slagboom PE , Snieder H , Spector TD , Thorsteinsdottir U , Stumvoll M , Tuomilehto J , Uitterlinden AG , Uusitupa M , van der Harst P , Veronesi G , Walker M , Wareham NJ , Watkins H , Wichmann HE , Abecasis GR , Assimes TL , Berndt SI , Boehnke M , Borecki IB , Deloukas P , Franke L , Frayling TM , Groop LC , Hunter DJ , Kaplan RC , O'Connell JR , Qi L , Schlessinger D , Strachan DP , Stefansson K , van Duijn CM , Willer CJ , Visscher PM , Yang J , Hirschhorn JN , Zillikens MC , McCarthy MI , Speliotes EK , North KE , Fox CS , Barroso I , Franks PW , Ingelsson E , Heid IM , Loos RJ , Cupples LA , Morris AP , Lindgren CM , Mohlke KL . New genetic loci link adipose and insulin biology to body fat distribution. Nature. 2015;518:187–196. 25673412 10.1038/nature14132 PMC4338562

[42] Willer CJ , Schmidt EM , Sengupta S , Peloso GM , Gustafsson S , Kanoni S , Ganna A , Chen J , Buchkovich ML , Mora S , Beckmann JS , Bragg‐Gresham JL , Chang HY , Demirkan A , Den Hertog HM , Do R , Donnelly LA , Ehret GB , Esko T , Feitosa MF , Ferreira T , Fischer K , Fontanillas P , Fraser RM , Freitag DF , Gurdasani D , Heikkila K , Hypponen E , Isaacs A , Jackson AU , Johansson A , Johnson T , Kaakinen M , Kettunen J , Kleber ME , Li X , Luan J , Lyytikainen LP , Magnusson PK , Mangino M , Mihailov E , Montasser ME , Muller‐Nurasyid M , Nolte IM , O'Connell JR , Palmer CD , Perola M , Petersen AK , Sanna S , Saxena R , Service SK , Shah S , Shungin D , Sidore C , Song C , Strawbridge RJ , Surakka I , Tanaka T , Teslovich TM , Thorleifsson G , Van den Herik EG , Voight BF , Volcik KA , Waite LL , Wong A , Wu Y , Zhang W , Absher D , Asiki G , Barroso I , Been LF , Bolton JL , Bonnycastle LL , Brambilla P , Burnett MS , Cesana G , Dimitriou M , Doney AS , Doring A , Elliott P , Epstein SE , Eyjolfsson GI , Gigante B , Goodarzi MO , Grallert H , Gravito ML , Groves CJ , Hallmans G , Hartikainen AL , Hayward C , Hernandez D , Hicks AA , Holm H , Hung YJ , Illig T , Jones MR , Kaleebu P , Kastelein JJ , Khaw KT , Kim E , Klopp N , Komulainen P , Kumari M , Langenberg C , Lehtimaki T , Lin SY , Lindstrom J , Loos RJ , Mach F , McArdle WL , Meisinger C , Mitchell BD , Muller G , Nagaraja R , Narisu N , Nieminen TV , Nsubuga RN , Olafsson I , Ong KK , Palotie A , Papamarkou T , Pomilla C , Pouta A , Rader DJ , Reilly MP , Ridker PM , Rivadeneira F , Rudan I , Ruokonen A , Samani N , Scharnagl H , Seeley J , Silander K , Stancakova A , Stirrups K , Swift AJ , Tiret L , Uitterlinden AG , van Pelt LJ , Vedantam S , Wainwright N , Wijmenga C , Wild SH , Willemsen G , Wilsgaard T , Wilson JF , Young EH , Zhao JH , Adair LS , Arveiler D , Assimes TL , Bandinelli S , Bennett F , Bochud M , Boehm BO , Boomsma DI , Borecki IB , Bornstein SR , Bovet P , Burnier M , Campbell H , Chakravarti A , Chambers JC , Chen YD , Collins FS , Cooper RS , Danesh J , Dedoussis G , de Faire U , Feranil AB , Ferrieres J , Ferrucci L , Freimer NB , Gieger C , Groop LC , Gudnason V , Gyllensten U , Hamsten A , Harris TB , Hingorani A , Hirschhorn JN , Hofman A , Hovingh GK , Hsiung CA , Humphries SE , Hunt SC , Hveem K , Iribarren C , Jarvelin MR , Jula A , Kahonen M , Kaprio J , Kesaniemi A , Kivimaki M , Kooner JS , Koudstaal PJ , Krauss RM , Kuh D , Kuusisto J , Kyvik KO , Laakso M , Lakka TA , Lind L , Lindgren CM , Martin NG , Marz W , McCarthy MI , McKenzie CA , Meneton P , Metspalu A , Moilanen L , Morris AD , Munroe PB , Njolstad I , Pedersen NL , Power C , Pramstaller PP , Price JF , Psaty BM , Quertermous T , Rauramaa R , Saleheen D , Salomaa V , Sanghera DK , Saramies J , Schwarz PE , Sheu WH , Shuldiner AR , Siegbahn A , Spector TD , Stefansson K , Strachan DP , Tayo BO , Tremoli E , Tuomilehto J , Uusitupa M , van Duijn CM , Vollenweider P , Wallentin L , Wareham NJ , Whitfield JB , Wolffenbuttel BH , Ordovas JM , Boerwinkle E , Palmer CN , Thorsteinsdottir U , Chasman DI , Rotter JI , Franks PW , Ripatti S , Cupples LA , Sandhu MS , Rich SS , Boehnke M , Deloukas P , Kathiresan S , Mohlke KL , Ingelsson E , Abecasis GR . Discovery and refinement of loci associated with lipid levels. Nat Genet. 2013;45:1274–1283. 24097068 10.1038/ng.2797 PMC3838666

[43] Arnold M , Raffler J , Pfeufer A , Suhre K , Kastenmuller G . Snipa: an interactive, genetic variant‐centered annotation browser. Bioinformatics. 2015;31:1334–1336. 25431330 10.1093/bioinformatics/btu779 PMC4393511

[44] Burgess S , Dudbridge F , Thompson SG . Combining information on multiple instrumental variables in Mendelian randomization: comparison of allele score and summarized data methods. Stat Med. 2016;35:1880–1906. 26661904 10.1002/sim.6835 PMC4832315

[45] Rosito GA , D'Agostino RB , Massaro J , Lipinska I , Mittleman MA , Sutherland P , Wilson PW , Levy D , Muller JE , Tofler GH . Association between obesity and a prothrombotic state: the Framingham Offspring Study. Thromb Haemost. 2004;91:683–689. 15045128 10.1160/th03-01-0014

[46] Coffey CS , Asselbergs FW , Hebert PR , Hillege HL , Li Q , Moore JH , van Gilst WH . The association of the metabolic syndrome with PAI‐1 and t‐PA levels. Cardiol Res Pract. 2011;2011:541467. 21559217 10.4061/2011/541467 PMC3087975

[47] Burgess S , Harshfield E . Mendelian randomization to assess causal effects of blood lipids on coronary heart disease: lessons from the past and applications to the future. Curr Opin Endocrinol Diabetes Obes. 2016;23:124–130. 26910273 10.1097/MED.0000000000000230 PMC4816855

[48] Do R , Willer CJ , Schmidt EM , Sengupta S , Gao C , Peloso GM , Gustafsson S , Kanoni S , Ganna A , Chen J , Buchkovich ML , Mora S , Beckmann JS , Bragg‐Gresham JL , Chang HY , Demirkan A , Den Hertog HM , Donnelly LA , Ehret GB , Esko T , Feitosa MF , Ferreira T , Fischer K , Fontanillas P , Fraser RM , Freitag DF , Gurdasani D , Heikkila K , Hypponen E , Isaacs A , Jackson AU , Johansson A , Johnson T , Kaakinen M , Kettunen J , Kleber ME , Li X , Luan J , Lyytikainen LP , Magnusson PK , Mangino M , Mihailov E , Montasser ME , Muller‐Nurasyid M , Nolte IM , O'Connell JR , Palmer CD , Perola M , Petersen AK , Sanna S , Saxena R , Service SK , Shah S , Shungin D , Sidore C , Song C , Strawbridge RJ , Surakka I , Tanaka T , Teslovich TM , Thorleifsson G , Van den Herik EG , Voight BF , Volcik KA , Waite LL , Wong A , Wu Y , Zhang W , Absher D , Asiki G , Barroso I , Been LF , Bolton JL , Bonnycastle LL , Brambilla P , Burnett MS , Cesana G , Dimitriou M , Doney AS , Doring A , Elliott P , Epstein SE , Eyjolfsson GI , Gigante B , Goodarzi MO , Grallert H , Gravito ML , Groves CJ , Hallmans G , Hartikainen AL , Hayward C , Hernandez D , Hicks AA , Holm H , Hung YJ , Illig T , Jones MR , Kaleebu P , Kastelein JJ , Khaw KT , Kim E , Klopp N , Komulainen P , Kumari M , Langenberg C , Lehtimaki T , Lin SY , Lindstrom J , Loos RJ , Mach F , McArdle WL , Meisinger C , Mitchell BD , Muller G , Nagaraja R , Narisu N , Nieminen TV , Nsubuga RN , Olafsson I , Ong KK , Palotie A , Papamarkou T , Pomilla C , Pouta A , Rader DJ , Reilly MP , Ridker PM , Rivadeneira F , Rudan I , Ruokonen A , Samani N , Scharnagl H , Seeley J , Silander K , Stancakova A , Stirrups K , Swift AJ , Tiret L , Uitterlinden AG , van Pelt LJ , Vedantam S , Wainwright N , Wijmenga C , Wild SH , Willemsen G , Wilsgaard T , Wilson JF , Young EH , Zhao JH , Adair LS , Arveiler D , Assimes TL , Bandinelli S , Bennett F , Bochud M , Boehm BO , Boomsma DI , Borecki IB , Bornstein SR , Bovet P , Burnier M , Campbell H , Chakravarti A , Chambers JC , Chen YD , Collins FS , Cooper RS , Danesh J , Dedoussis G , de Faire U , Feranil AB , Ferrieres J , Ferrucci L , Freimer NB , Gieger C , Groop LC , Gudnason V , Gyllensten U , Hamsten A , Harris TB , Hingorani A , Hirschhorn JN , Hofman A , Hovingh GK , Hsiung CA , Humphries SE , Hunt SC , Hveem K , Iribarren C , Jarvelin MR , Jula A , Kahonen M , Kaprio J , Kesaniemi A , Kivimaki M , Kooner JS , Koudstaal PJ , Krauss RM , Kuh D , Kuusisto J , Kyvik KO , Laakso M , Lakka TA , Lind L , Lindgren CM , Martin NG , Marz W , McCarthy MI , McKenzie CA , Meneton P , Metspalu A , Moilanen L , Morris AD , Munroe PB , Njolstad I , Pedersen NL , Power C , Pramstaller PP , Price JF , Psaty BM , Quertermous T , Rauramaa R , Saleheen D , Salomaa V , Sanghera DK , Saramies J , Schwarz PE , Sheu WH , Shuldiner AR , Siegbahn A , Spector TD , Stefansson K , Strachan DP , Tayo BO , Tremoli E , Tuomilehto J , Uusitupa M , van Duijn CM , Vollenweider P , Wallentin L , Wareham NJ , Whitfield JB , Wolffenbuttel BH , Altshuler D , Ordovas JM , Boerwinkle E , Palmer CN , Thorsteinsdottir U , Chasman DI , Rotter JI , Franks PW , Ripatti S , Cupples LA , Sandhu MS , Rich SS , Boehnke M , Deloukas P , Mohlke KL , Ingelsson E , Abecasis GR , Daly MJ , Neale BM , Kathiresan S . Common variants associated with plasma triglycerides and risk for coronary artery disease. Nat Genet. 2013;45:1345–1352. 24097064 10.1038/ng.2795 PMC3904346

[49] Eriksson P , Kallin B , van ‘t Hooft FM , Bavenholm P , Hamsten A . Allele‐specific increase in basal transcription of the plasminogen‐activator inhibitor 1 gene is associated with myocardial infarction. Proc Natl Acad Sci USA. 1995;92:1851–1855. 7892190 10.1073/pnas.92.6.1851 PMC42380

[50] Beier JI , Kaiser JP , Guo L , Martinez‐Maldonado M , Arteel GE . Plasminogen activator inhibitor‐1 deficient mice are protected from angiotensin II‐induced fibrosis. Arch Biochem Biophys. 2011;510:19–26. 21501583 10.1016/j.abb.2011.04.001 PMC3095665

[51] Ingelsson E , Pencina MJ , Tofler GH , Benjamin EJ , Lanier KJ , Jacques PF , Fox CS , Meigs JB , Levy D , Larson MG , Selhub J , D'Agostino RB Sr , Wang TJ , Vasan RS . Multimarker approach to evaluate the incidence of the metabolic syndrome and longitudinal changes in metabolic risk factors: the Framingham Offspring Study. Circulation. 2007;116:984–992. 17698726 10.1161/CIRCULATIONAHA.107.708537

[52] Heldgaard PE , Sidelmann JJ , Hindsberger C , Olivarius Nde F , Henriksen JE , Gram J . Relationship of glucose concentrations with PAI‐1 and t‐PA in subjects with normal glucose tolerance. Diabet Med. 2006;23:887–893. 16911627 10.1111/j.1464-5491.2006.01924.x

[53] Tamura Y , Kawao N , Yano M , Okada K , Matsuo O , Kaji H . Plasminogen activator inhibitor‐1 deficiency ameliorates insulin resistance and hyperlipidemia but not bone loss in obese female mice. Endocrinology. 2014;155:1708–1717. 24605827 10.1210/en.2013-1888

[54] Crandall DL , Quinet EM , El Ayachi S , Hreha AL , Leik CE , Savio DA , Juhan‐Vague I , Alessi MC . Modulation of adipose tissue development by pharmacological inhibition of PAI‐1. Arterioscler Thromb Vasc Biol. 2006;26:2209–2215. 16825598 10.1161/01.ATV.0000235605.51400.9d

[55] Crandall DL , Busler DE , McHendry‐Rinde B , Groeling TM , Kral JG . Autocrine regulation of human preadipocyte migration by plasminogen activator inhibitor‐1. J Clin Endocrinol Metab. 2000;85:2609–2614. 10902815 10.1210/jcem.85.7.6678

[56] Halleux CM , Declerck PJ , Tran SL , Detry R , Brichard SM . Hormonal control of plasminogen activator inhibitor‐1 gene expression and production in human adipose tissue: stimulation by glucocorticoids and inhibition by catecholamines. J Clin Endocrinol Metab. 1999;84:4097–4105. 10566656 10.1210/jcem.84.11.6127

[57] Holmes MV , Asselbergs FW , Palmer TM , Drenos F , Lanktree MB , Nelson CP , Dale CE , Padmanabhan S , Finan C , Swerdlow DI , Tragante V , van Iperen EP , Sivapalaratnam S , Shah S , Elbers CC , Shah T , Engmann J , Giambartolomei C , White J , Zabaneh D , Sofat R , McLachlan S ; Consortium U , Doevendans PA , Balmforth AJ , Hall AS , North KE , Almoguera B , Hoogeveen RC , Cushman M , Fornage M , Patel SR , Redline S , Siscovick DS , Tsai MY , Karczewski KJ , Hofker MH , Verschuren WM , Bots ML , van der Schouw YT , Melander O , Dominiczak AF , Morris R , Ben‐Shlomo Y , Price J , Kumari M , Baumert J , Peters A , Thorand B , Koenig W , Gaunt TR , Humphries SE , Clarke R , Watkins H , Farrall M , Wilson JG , Rich SS , de Bakker PI , Lange LA , Davey Smith G , Reiner AP , Talmud PJ , Kivimaki M , Lawlor DA , Dudbridge F , Samani NJ , Keating BJ , Hingorani AD , Casas JP . Mendelian randomization of blood lipids for coronary heart disease. Eur Heart J. 2015;36:539–550. 24474739 10.1093/eurheartj/eht571 PMC4344957

[58] Zanoni P , Khetarpal SA , Larach DB , Hancock‐Cerutti WF , Millar JS , Cuchel M , DerOhannessian S , Kontush A , Surendran P , Saleheen D , Trompet S , Jukema JW , De Craen A , Deloukas P , Sattar N , Ford I , Packard C , Majumder A , Alam DS , Di Angelantonio E , Abecasis G , Chowdhury R , Erdmann J , Nordestgaard BG , Nielsen SF , Tybjaerg‐Hansen A , Schmidt RF , Kuulasmaa K , Liu DJ , Perola M , Blankenberg S , Salomaa V , Mannisto S , Amouyel P , Arveiler D , Ferrieres J , Muller‐Nurasyid M , Ferrario M , Kee F , Willer CJ , Samani N , Schunkert H , Butterworth AS , Howson JM , Peloso GM , Stitziel NO , Danesh J , Kathiresan S , Rader DJ ; Consortium CHDE, Consortium CAE, Global Lipids Genetics C . Rare variant in scavenger receptor BI raises HDL cholesterol and increases risk of coronary heart disease. Science. 2016;351:1166–1171. 26965621 10.1126/science.aad3517 PMC4889017

