# Supplementary material for: Causal Effect of Plasminogen Activator Inhibitor Type 1 on Coronary Heart Disease
Source: J Am Heart Assoc. 2017 May 26;6(6):e004918. doi: 10.1161/JAHA.116.004918 (PMC5669150; doi:10.1161/JAHA.116.004918)
Supplement: Supplementary file 1 — Data S1. Table S1. Publications Included in the Observational Meta‐Analysis Table S2. SNPs Involved in the Genetic Risk Scores as Instrumental Variable for PAI‐1 Table S3. Causal Effect of BMI on PAI‐1 [file JAH3-6-e004918-s001.pdf]

Data S1

Methods for causal effect of BMI on PAI-1

To further investigate our findings on the negative direction of effect of PAI-1 on BMI, which runs counter to some prior evidence,[1] we explored the causal effect of BMI on PAI-1. Locke *et al.* reported 77 independent genome-wide significant loci associated with BMI in 322,154 individuals of European ancestry.[2] We applied the MR approach using all 77 loci as an instrumental variable (IV) using the inverse variance weighted (IVW) method described in our main article. Two sensitivity analyses were further adopted to examine potential pleiotropic effects of the selected IV. In the first sensitivity analysis, we used the MR-Egger method.[3] In this method, an Egger regression, which is commonly used to detect small study bias, was used to detect the potential bias of pleiotropic effects in the MR. The beta coefficient in the MR-Egger is considered as a causal effect after correcting for pleiotropic effects. The intercept of the Egger regression provides information on the directional pleiotropic effect in the IV (right panel in **Table S3**).[3] In the second sensitivity analysis, we investigated whether the causal effect was consistent when only 50% of the SNPs are assumed to be valid in the IV, an approach known as weighted median estimator.[4]

Results for causal effect of BMI on PAI-1

We found that increasing 1 unit of BMI was causally associated with a 0.21 unit increase of log-transformed PAI-1 (beta, 0.21, 95% CI, 0.13, 0.29; **Table S3**). The result was consistent when the MR-Egger method was applied (beta, 0.21, 95% CI, 0.02, 0.41; **Table S3**), with little influence of pleiotropic effect as indicated by the intercept (beta, -0.0002, P-value, 0.949). The median estimator was also in agreement with the other two analyses (beta, 0.22, 95% CI, 0.09, 0.36; **Table S3**). Taken together, our results support a robust positive causal effect of BMI on PAI-1.

Table S1. Publications included in the observational meta-analysis

Study	PMID	Adjustment in the multiple-variable model
Thögersen *et al*, 1998[5]	9826309	Age, sex, diabetes, smoking, hypertension, BMI, Cholesterol, and ApoA-1.
Cushman *et al*, 1999[6]	10073948	Hypertension, smoking status, race (white or nonwhite), diabetes, and body mass index.
Söderberg *et al*, 1999[7]	10583712	BMI, hypertension, history of diabetes, daily smoking habits, cholesterol levels, leptin, apo A-1, apo B, and insulin.
Folsom *et al*, 2001[8]	11304480	Age, race, and sex, smoking status (never, former, current), total cholesterol, HDL cholesterol, systolic blood pressure, use of antihypertensive medication, and diabetes.
Thøgersen *et al*, 2004[9]	15167204	N/A (dichotomous analysis).
Smith *et al*, 2005[10]	16286603	Age, sex, race, hypertension, diabetes mellitus, total cholesterol, HDL, cigarette smoking and alcohol intake.
Aleksic *et al*, 2009[11]	18342864	Age, sex, race, hypertension, diabetes mellitus, total cholesterol, HDL, cigarette smoking and alcohol intake.
Thøgersen *et al*, 2009[12]	19357504	Age, smoking, CRP, t-PA, creatinine.
Luc *et al*, 2010[13]	19823188	Diabetes, hypertension, smoking status, total and high-density lipoprotein (HDL) cholesterol and triglycerides.
Meltzer *et al*, 2010[14]	20413657	Age, HDL and total cholesterol, triglycerides, BMI, and diabetes.
Yano *et al*, 2013[15]	23551722	Sex, body mass index, history of diabetes, history of hyperlipidemia, and 24-hour pulse rate, high levels of high-sensitivity C-reactive protein, prothrombin fragment 1+2.
De Luca *et al*, 2013[16]	24004495	Duration of time between the date of samples and the analysis time, age, total cholesterol, HDL.

Knudsen *et al*, 2014[17]	24566095	Viral load and a high (Data collection on Adverse events of Anti-HIV Drugs) D:A:D risk score.
Tofler *et al*, 2016[18]	26896607	Age, sex, systolic blood pressure, anti-hypertensive therapy, BMI, diabetes, cigarette smoking, total cholesterol, HDL cholesterol, and triglycerides.

Table S2. SNPs involved in the genetic risk scores as instrumental variable for PAI-1

SNPid	Chr:position(hg19)	Risk/Other alleles	Allele freq in PAI-1*	Allele freq in CHD*	Effect on PAI-1**	Effect on CHD***
SNPs involved in the single locus instrumental variable						
rs2227631	chr7:100,769,538	A/G	0.592	0.563	0.076 (0.010)	0.008 (0.009)
rs2075756	chr7:100,466,441	A/G	0.282	0.279	0.058 (0.010)	0.027 (0.010)
rs12672665	chr7:100,483,731	A/G	0.479	0.474	0.047 (0.009)	0.012 (0.009)
rs757718	chr7:100,792,810	T/C	0.093	0.118	0.081 (0.016)	-0.013 (0.017)
SNPs involved in the multi locus instrumental variable						
rs2227631	chr7:100,769,538	A/G	0.592	0.563	0.076 (0.010)	0.008 (0.009)
rs6976053	chr7:100,512,119	T/C	0.479	0.476	0.054 (0.009)	0.019 (0.009)
rs6486122	chr11:13,361,524	T/C	0.689	0.638	0.051 (0.009)	0.034 (0.010)
rs11128603	chr3:12,385,828	A/G	0.898	0.870	0.086 (0.016)	-0.001 (0.014)

* Allele freq in PAI-1 and CHD reported the allele frequency of the risk allele in the study samples for PAI-1 and CHD respectively.

** Effect of SNPs on PAI-1 is reported as beta coefficient (with standard error) from Huang *et al*, 2012.[19]

*** Effect of SNPs on CHD is reported as log-transformed Odds ratio (with standard error) from Nikpay *et al*, 2015.[20]

Table S3. Causal effect of BMI on PAI-1

	Causal effect of BMI on PAI-1			Directional pleiotropic effect	
Method	Beta	95% CI	P-value	Beta	P-value
Primary MR analysis					
IVW approach	0.21	0.13, 0.29	4.31E-07	N/A	N/A
Sensitivity analysis					
MR_Egger	0.21	0.02, 0.41	0.034	-0.0002	0.949
Weighted median estimator	0.22	0.09, 0.36	0.001	N/A	N/A

Supplementary References

1. Ingelsson E, Pencina MJ, Tofler GH, Benjamin EJ, Lanier KJ, Jacques PF, Fox CS, Meigs JB, Levy D, Larson MG, Selhub J, D'Agostino RB, Sr., Wang TJ, Vasan RS. Multimarker approach to evaluate the incidence of the metabolic syndrome and longitudinal changes in metabolic risk factors: The framingham offspring study. *Circulation*. 2007;116:984-992
2. Locke AE, Kahali B, Berndt SI, Justice AE, Pers TH, Day FR, Powell C, Vedantam S, Buchkovich ML, Yang J, Croteau-Chonka DC, Esko T, Fall T, Ferreira T, Gustafsson S, Kutalik Z, Luan J, Magi R, Randall JC, Winkler TW, Wood AR, Workalemahu T, Faul JD, Smith JA, Hua Zhao J, Zhao W, Chen J, Fehrmann R, Hedman AK, Karjalainen J, Schmidt EM, Absher D, Amin N, Anderson D, Beekman M, Bolton JL, Bragg-Gresham JL, Buyske S, Demirkan A, Deng G, Ehret GB, Feenstra B, Feitosa MF, Fischer K, Goel A, Gong J, Jackson AU, Kanoni S, Kleber ME, Kristiansson K, Lim U, Lotay V, Mangino M, Mateo Leach I, Medina-Gomez C, Medland SE, Nalls MA, Palmer CD, Pasko D, Pechlivanis S, Peters MJ, Prokopenko I, Shungin D, Stancakova A, Strawbridge RJ, Ju Sung Y, Tanaka T, Teumer A, Trompet S, van der Laan SW, van Setten J, Van Vliet-Ostaptchouk JV, Wang Z, Yengo L, Zhang W, Isaacs A, Albrecht E, Arnlov J, Arscott GM, Attwood AP, Bandinelli S, Barrett A, Bas IN, Bellis C, Bennett AJ, Berne C, Blagieva R, Bluher M, Bohringer S, Bonnycastle LL, Bottcher Y, Boyd HA, Bruinenberg M, Caspersen IH, Ida Chen YD, Clarke R, Daw EW, de Craen AJ, Delgado G, Dimitriou M, Doney AS, Eklund N, Estrada K, Eury E, Folkersen L, Fraser RM, Garcia ME, Geller F, Giedraitis V, Gigante B, Go AS, Golay A, Goodall AH, Gordon SD, Gorski M, Grabe HJ, Grallert H, Grammer TB, Grassler J, Gronberg H, Groves CJ, Gusto G, Haessler J, Hall P, Haller T, Hallmans G, Hartman CA, Hassinen M, Hayward C, Heard-Costa NL, Helmer Q, Hengstenberg C, Holmen O, Hottenga JJ, James AL, Jeff JM, Johansson A, Jolley J, Juliusdottir T, Kinnunen L, Koenig W, Koskenvuo M, Kratzer W, Laitinen J, Lamina C, Leander K, Lee NR, Lichtner P, Lind L, Lindstrom J, Sin Lo K, Lobbens S, Lorbeer R, Lu Y, Mach F, Magnusson PK, Mahajan A, McArdle WL, McLachlan S, Menni C, Merger S, Mihailov E, Milani L, Moayyeri A, Monda KL, Morken MA, Mulas A, Muller G, Muller-Nurasyid M, Musk AW, Nagaraja R, Nothen MM, Nolte IM, Pilz S, Rayner NW, Renstrom F, Rettig R, Ried JS, Ripke S, Robertson NR, Rose LM, Sanna S, Scharnagl H, Scholtens S, Schumacher FR, Scott WR, Seufferlein T, Shi J, Vernon Smith A, Smolonska J, Stanton AV, Steinthorsdottir V, Stirrups K, Stringham HM, Sundstrom J, Swertz MA, Swift AJ, Syvanen AC, Tan ST, Tayo BO, Thorand B, Thorleifsson G, Tyrer JP, Uh HW, Vandenput L, Verhulst FC, Vermeulen SH, Verweij N, Vonk JM, Waite LL, Warren HR, Waterworth D, Weedon MN, Wilkens LR, Willenborg C, Wilsgaard T, Wojczynski MK, Wong A, Wright AF, Zhang Q, LifeLines Cohort S, Brennan EP, Choi M, Dastani Z, Drong AW, Eriksson P, Franco-Cereceda A, Gadin JR, Gharavi AG, Goddard ME, Handsaker RE, Huang J, Karpe F, Kathiresan S, Keildson S, Kiryluk K, Kubo M, Lee JY, Liang L, Lifton RP, Ma B, McCarroll SA, McKnight AJ, Min JL, Moffatt MF, Montgomery GW, Murabito JM, Nicholson G, Nyholt DR, Okada Y, Perry JR, Dorajoo R, Reinmaa E, Salem RM, Sandholm N, Scott RA, Stolk L, Takahashi A, Tanaka T, Van't Hooft FM, Vinkhuyzen AA, Westra HJ, Zheng W, Zondervan KT, Consortium AD, Group A-BW, Consortium CAD, Consortium CK, Glgc, Icbp, Investigators M, Mu TC, Consortium MI, Consortium P, ReproGen C, Consortium G, International Endogene C, Heath AC, Arveiler D, Bakker SJ, Beilby J, Bergman RN, Blangero J, Bovet P, Campbell H, Caulfield MJ, Cesana G, Chakravarti A, Chasman DI, Chines PS, Collins FS, Crawford DC, Cupples LA, Cusi D, Danesh J, de Faire U, den Ruijter HM, Dominiczak AF, Erbel R, Erdmann J, Eriksson JG, Farrall M, Felix SB, Ferrannini E, Ferrieres J, Ford I, Forouhi NG, Forrester T, Franco OH, Gansevoort RT, Gejman PV, Gieger C, Gottesman O, Gudnason V, Gyllensten U, Hall AS, Harris TB, Hattersley AT, Hicks AA, Hindorff LA, Hingorani AD, Hofman A, Homuth G, Hovingh GK, Humphries SE, Hunt SC, Hypponen E, Illig T, Jacobs KB, Jarvelin MR, Jockel KH, Johansen B, Jousilahti P, Jukema JW, Jula AM, Kaprio J, Kastelein JJ,

Keinanen-Kiukaanniemi SM, Kiemeney LA, Knekt P, Kooner JS, Kooperberg C, Kovacs P, Kraja AT, Kumari M, Kuusisto J, Lakka TA, Langenberg C, Le Marchand L, Lehtimaki T, Lyssenko V, Mannisto S, Marette A, Matise TC, McKenzie CA, McKnight B, Moll FL, Morris AD, Morris AP, Murray JC, Nelis M, Ohlsson C, Oldehinkel AJ, Ong KK, Madden PA, Pasterkamp G, Peden JF, Peters A, Postma DS, Pramstaller PP, Price JF, Qi L, Raitakari OT, Rankinen T, Rao DC, Rice TK, Ridker PM, Rioux JD, Ritchie MD, Rudan I, Salomaa V, Samani NJ, Saramies J, Sarzynski MA, Schunkert H, Schwarz PE, Sever P, Shuldiner AR, Sinisalo J, Stolk RP, Strauch K, Tonjes A, Tregouet DA, Tremblay A, Tremoli E, Virtamo J, Vohl MC, Volker U, Waeber G, Willemsen G, Witteman JC, Zillikens MC, Adair LS, Amouyel P, Asselbergs FW, Assimes TL, Bochud M, Boehm BO, Boerwinkle E, Bornstein SR, Bottinger EP, Bouchard C, Cauchi S, Chambers JC, Chanock SJ, Cooper RS, de Bakker PI, Dedoussis G, Ferrucci L, Franks PW, Froguel P, Groop LC, Haiman CA, Hamsten A, Hui J, Hunter DJ, Hveem K, Kaplan RC, Kivimaki M, Kuh D, Laakso M, Liu Y, Martin NG, Marz W, Melbye M, Metspalu A, Moebus S, Munroe PB, Njolstad I, Oostra BA, Palmer CN, Pedersen NL, Perola M, Perusse L, Peters U, Power C, Quertermous T, Rauramaa R, Rivadeneira F, Saaristo TE, Saleheen D, Sattar N, Schadt EE, Schlessinger D, Slagboom PE, Snieder H, Spector TD, Thorsteinsdottir U, Stumvoll M, Tuomilehto J, Uitterlinden AG, Uusitupa M, van der Harst P, Walker M, Wallaschofski H, Wareham NJ, Watkins H, Weir DR, Wichmann HE, Wilson JF, Zanen P, Borecki IB, Deloukas P, Fox CS, Heid IM, O'Connell JR, Strachan DP, Stefansson K, van Duijn CM, Abecasis GR, Franke L, Frayling TM, McCarthy MI, Visscher PM, Scherag A, Willer CJ, Boehnke M, Mohlke KL, Lindgren CM, Beckmann JS, Barroso I, North KE, Ingelsson E, Hirschhorn JN, Loos RJ, Speliotes EK. Genetic studies of body mass index yield new insights for obesity biology. *Nature*. 2015;518:197-206

3. Bowden J, Davey Smith G, Burgess S. Mendelian randomization with invalid instruments: Effect estimation and bias detection through egger regression. *International journal of epidemiology*. 2015;44:512-525
4. Bowden J, Davey Smith G, Haycock PC, Burgess S. Consistent estimation in mendelian randomization with some invalid instruments using a weighted median estimator. *Genetic epidemiology*. 2016;40:304-314
5. Thogersen AM, Jansson JH, Boman K, Nilsson TK, Weinehall L, Huhtasaari F, Hallmans G. High plasminogen activator inhibitor and tissue plasminogen activator levels in plasma precede a first acute myocardial infarction in both men and women: Evidence for the fibrinolytic system as an independent primary risk factor. *Circulation*. 1998;98:2241-2247
6. Cushman M, Lemaitre RN, Kuller LH, Psaty BM, Macy EM, Sharrett AR, Tracy RP. Fibrinolytic activation markers predict myocardial infarction in the elderly. The cardiovascular health study. *Arteriosclerosis, thrombosis, and vascular biology*. 1999;19:493-498
7. Soderberg S, Ahren B, Jansson JH, Johnson O, Hallmans G, Asplund K, Olsson T. Leptin is associated with increased risk of myocardial infarction. *Journal of internal medicine*. 1999;246:409-418
8. Folsom AR, Aleksic N, Park E, Salomaa V, Juneja H, Wu KK. Prospective study of fibrinolytic factors and incident coronary heart disease: The atherosclerosis risk in communities (aric) study. *Arteriosclerosis, thrombosis, and vascular biology*. 2001;21:611-617
9. Thogersen AM, Soderberg S, Jansson JH, Dahlen G, Boman K, Nilsson TK, Lindahl B, Weinehall L, Stenlund H, Lundberg V, Johnson O, Ahren B, Hallmans G. Interactions between fibrinolysis, lipoproteins and leptin related to a first myocardial infarction. *European journal of cardiovascular prevention and rehabilitation : official journal of the European Society of Cardiology, Working Groups on Epidemiology & Prevention and Cardiac Rehabilitation and Exercise Physiology*. 2004;11:33-40

10. Smith A, Patterson C, Yarnell J, Rumley A, Ben-Shlomo Y, Lowe G. Which hemostatic markers add to the predictive value of conventional risk factors for coronary heart disease and ischemic stroke? The caerphilly study. *Circulation*. 2005;112:3080-3087
11. Aleksic N, Wang YW, Ahn C, Juneja HS, Folsom AR, Wu KK. Assessment of coronary heart disease risk by combined analysis of coagulation factors. *Atherosclerosis*. 2008;198:294-300
12. Thogersen AM, Nilsson TK, Weinehall L, Boman K, Eliasson M, Hallmans G, Jansson JH. Changes in plasma c-reactive protein and hemostatic factors prior to and after a first myocardial infarction with a median follow-up time of 8 years. *Blood coagulation & fibrinolysis : an international journal in haemostasis and thrombosis*. 2009;20:340-346
13. Luc G, Empana JP, Morange P, Juhan-Vague I, Arveiler D, Ferrieres J, Amouyel P, Evans A, Kee F, Bingham A, Machez E, Ducimetiere P. Adipocytokines and the risk of coronary heart disease in healthy middle aged men: The prime study. *International journal of obesity*. 2010;34:118-126
14. Meltzer ME, Doggen CJ, de Groot PG, Rosendaal FR, Lisman T. Plasma levels of fibrinolytic proteins and the risk of myocardial infarction in men. *Blood*. 2010;116:529-536
15. Yano Y, Hoshide S, Shimada K, Kario K. The impact of cigarette smoking on 24-hour blood pressure, inflammatory and hemostatic activity, and cardiovascular risk in japanese hypertensive patients. *Journal of clinical hypertension*. 2013;15:234-240
16. De Luca A, de Gaetano Donati K, Colafigli M, Cozzi-Lepri A, De Curtis A, Gori A, Sighinolfi L, Giacometti A, Capobianchi MR, D'Avino A, Iacoviello L, Cauda R, D'Arminio Monforte A. The association of high-sensitivity c-reactive protein and other biomarkers with cardiovascular disease in patients treated for hiv: A nested case-control study. *BMC infectious diseases*. 2013;13:414
17. Knudsen A, Katzenstein TL, Benfield T, Jorgensen NR, Kronborg G, Gerstoft J, Obel N, Kjaer A, Lebech AM. Plasma plasminogen activator inhibitor-1 predicts myocardial infarction in hiv-1-infected individuals. *Aids*. 2014;28:1171-1179
18. Tofler GH, Massaro J, O'Donnell CJ, Wilson PW, Vasan RS, Sutherland PA, Meigs JB, Levy D, D'Agostino RB, Sr. Plasminogen activator inhibitor and the risk of cardiovascular disease: The framingham heart study. *Thrombosis research*. 2016;140:30-35
19. Huang J, Sabater-Lleal M, Asselbergs FW, Tregouet D, Shin SY, Ding J, Baumert J, Oudot-Mellakh T, Folkersen L, Johnson AD, Smith NL, Williams SM, Ikram MA, Kleber ME, Becker DM, Truong V, Mychaleckyj JC, Tang W, Yang Q, Sennblad B, Moore JH, Williams FM, Dehghan A, Silbernagel G, Schrijvers EM, Smith S, Karakas M, Tofler GH, Silveira A, Navis GJ, Lohman K, Chen MH, Peters A, Goel A, Hopewell JC, Chambers JC, Saleheen D, Lundmark P, Psaty BM, Strawbridge RJ, Boehm BO, Carter AM, Meisinger C, Peden JF, Bis JC, McKnight B, Ohrvik J, Taylor K, Franzosi MG, Seedorf U, Collins R, Franco-Cereceda A, Syvanen AC, Goodall AH, Yanek LR, Cushman M, Muller-Nurasyid M, Folsom AR, Basu S, Matijevic N, van Gilst WH, Kooner JS, Hofman A, Danesh J, Clarke R, Meigs JB, Consortium D, Kathiresan S, Reilly MP, Consortium CA, Klopp N, Harris TB, Winkelmann BR, Grant PJ, Hillege HL, Watkins H, Consortium CD, Spector TD, Becker LC, Tracy RP, Marz W, Uitterlinden AG, Eriksson P, Cambien F, Consortium C, Morange PE, Koenig W, Soranzo N, van der Harst P, Liu Y, O'Donnell CJ, Hamsten A. Genome-wide association study for circulating levels of pai-1 provides novel insights into its regulation. *Blood*. 2012;120:4873-4881
20. Nikpay M, Goel A, Won HH, Hall LM, Willenborg C, Kanoni S, Saleheen D, Kyriakou T, Nelson CP, Hopewell JC, Webb TR, Zeng L, Dehghan A, Alver M, Armasu SM, Auro K, Bjonnes A, Chasman DI, Chen S, Ford I, Franceschini N, Gieger C, Grace C, Gustafsson S, Huang J, Hwang SJ, Kim YK, Kleber ME, Lau KW, Lu X, Lu Y, Lyytikainen LP, Mihailov E, Morrison AC, Pervjakova N, Qu L, Rose LM, Salfati E, Saxena R, Scholz M, Smith AV, Tikkanen E, Uitterlinden A, Yang X, Zhang W, Zhao W, de Andrade M, de Vries PS, van Zuydam NR, Anand SS, Bertram L, Beutner F, Dedoussis G, Frossard P, Gauguier D, Goodall AH, Gottesman O, Haber M, Han BG, Huang J, Jalilzadeh S,

Kessler T, Konig IR, Lannfelt L, Lieb W, Lind L, Lindgren CM, Lokki ML, Magnusson PK, Mallick NH, Mehra N, Meitinger T, Memon FU, Morris AP, Nieminen MS, Pedersen NL, Peters A, Rallidis LS, Rasheed A, Samuel M, Shah SH, Sinisalo J, Stirrups KE, Trompet S, Wang L, Zaman KS, Ardissino D, Boerwinkle E, Borecki IB, Bottinger EP, Buring JE, Chambers JC, Collins R, Cupples LA, Danesh J, Demuth I, Elosua R, Epstein SE, Esko T, Feitosa MF, Franco OH, Franzosi MG, Granger CB, Gu D, Gudnason V, Hall AS, Hamsten A, Harris TB, Hazen SL, Hengstenberg C, Hofman A, Ingelsson E, Iribarren C, Jukema JW, Karhunen PJ, Kim BJ, Kooner JS, Kullo IJ, Lehtimaki T, Loos RJ, Melander O, Metspalu A, Marz W, Palmer CN, Perola M, Quertermous T, Rader DJ, Ridker PM, Ripatti S, Roberts R, Salomaa V, Sanghera DK, Schwartz SM, Seedorf U, Stewart AF, Stott DJ, Thiery J, Zalloua PA, O'Donnell CJ, Reilly MP, Assimes TL, Thompson JR, Erdmann J, Clarke R, Watkins H, Kathiresan S, McPherson R, Deloukas P, Schunkert H, Samani NJ, Farrall M, Consortium CAD. A comprehensive 1,000 genomes-based genome-wide association meta-analysis of coronary artery disease. *Nature genetics*. 2015;47:1121-1130
